# Verdazyls as Possible Building Blocks for Multifunctional Molecular Materials: A Case Study on 1,5-Diphenyl-3-(*p-*iodophenyl)-verdazyl Focusing on Magnetism, Electron Transfer and the Applicability of the Sonogashira-Hagihara Reaction

**DOI:** 10.3390/molecules23071758

**Published:** 2018-07-18

**Authors:** Hannah Jobelius, Norbert Wagner, Gregor Schnakenburg, Andreas Meyer

**Affiliations:** 1 Institute of Physical and Theoretical Chemistry, University of Bonn, 53115 Bonn, Germany; hannah.jobelius@uni-bonn.de; 2 Institute of Inorganic Chemistry, University of Bonn, 53121 Bonn, Germany; unc40056@uni-bonn.de (N.W.); gregor.schnakenburg@uni-bonn.de (G.S.); 3 Max-Planck-Institute for Biophysical Chemistry, 37077 Göttingen, Germany

**Keywords:** radicals, exchange coupling, cross-coupling, broken symmetry DFT, EPR spectroscopy, X-ray crystallography, redox chemistry

## Abstract

This work explores the use of Kuhn verdazyl radicals as building blocks in multifunctional molecular materials in an exemplary study, focusing on the magnetic and the electron transfer (ET) characteristics, but also addressing the question whether chemical modification by cross-coupling is possible. The ET in solution is studied spectroscopically, whereas solid state measurements afford information about the magnetic susceptibility or the conductivity of the given samples. The observed results are rationalized based on the chemical structures of the molecules, which have been obtained by X-ray crystallography. The crystallographically observed molecular structures as well as the interpretation based on the spectroscopic and physical measurements are backed up by DFT calculations. The measurements indicate that only weak, antiferromagnetic (AF) coupling is observed in Kuhn verdazyls owed to the low tendency to form face-to-face stacks, but also that steric reasons alone are not sufficient to explain this behavior. Furthermore, it is also demonstrated that ET reactions proceed rapidly in verdazyl/verdazylium redox couples and that Kuhn verdazyls are suited as donor molecules in ET reactions.

## 1. Introduction

**Figure 1:**
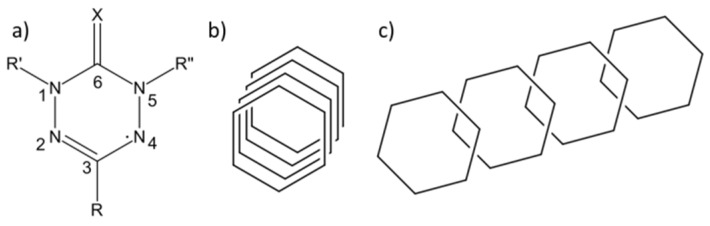
(**a**) Structure of Kuhn verdazyl (X = 2H) and oxoverdazyl (X = O) radicals. (**b**) Schematic representation of face-to-face stacking of hexagonal rings without slippage. (**c**) Slipped stacking.

The design of multifunctional molecular materials (MMM) employing organic radicals as fundamental building blocks is an active area of research [1]. Organic radicals are useful building blocks because of their intrinsic magnetic properties [2,3] and their ability to partake in electron transfer (ET) reactions as both electron acceptor or donor [4]. Furthermore, it is possible to synthesize molecules with tailormade properties by introducing suited substituents into the molecular backbone of the radical moiety, including the attachment of optically active groups or further spin centers [5,6,7]. To be suited as a building block in MMM, the radical compound needs to be sufficiently stable to allow usage under practical conditions, ideally at room temperature and in contact with air. The most prominent families of radicals that are stable under these conditions are certainly nitroxides and nitronyl-nitroxides, which have been known and established for about half a century and have found widespread use in different fields [8,9,10,11,12]. Another class of nitrogen-centered radicals is formed by the Kuhn verdazyl and the oxoverdazyl radical families (Figure 1a) [13,14].

Despite being discovered almost as early as the nitroxide based radicals, verdazyl radicals have not been investigated as thoroughly. However, the continued effort to extend the pool of building blocks for MMM has led to renewed interest in the physics and chemistry of verdazyls. Among the physical properties of verdazyl radicals, their solid state magnetism has received the most attention [15]. Strong magnetic interactions between verdazyl molecules are observed in cases where the heterocyclic planes of two verdazyls are arranged in a stacked manner, with reduced exchange coupling constants when the planes form slipped stacks (Figure 1b,c). Accordingly, the strongest interactions were observed in a bis-verdazyl substituted ferrocene, where the stacking in the solid state was brought about by the template effect of the ferrocene moiety (estimated, antiferromagnetic (AF) coupling constant *J* > 2000 cm^−1^) [16], and in an imidazolyl substituted verdazyl, where efficient stacking was achieved after ligation to a Cu(I) center (calculated *J* of ~1500 cm^−1^ at 120 K) [17]. In cases where efficient stacking in the crystalline state occurred, rather strong AF couplings have been observed in purely organic, metal-free compounds as well [18,19,20,21,22]. One example of a purely organic verdazyl compound with stacked structure exhibiting ferromagnetic coupling was presented in 2014 by Mehri et al. [23]. Noteworthy, three of the cited examples of purely organic compounds in which effective stacking was observed contain acetylene or substituted acetylene moieties [21,22,23]. Aside from these experimental studies, computational studies concerned with either the pairwise interaction between verdazyl radicals [24] or the bulk interaction in the actual solid state structure of a given verdazyl have been presented [25,26].

Combining the magnetism of verdazyls with electron transport would open up interesting applications of verdazyl radicals in the field of spintronics [27,28,29]. As demonstrated by the Pilkington group, one way to obtain molecular building blocks with these two properties combined is the covalent attachment of tetrathiafulvalene (TTF) groups to a verdazyl radical [30,31,32,33]. In these compounds, the possibility of ET is provided by the TTF groups, whereas the verdazyl radical provides the magnetism. However, owed to their radical character, the verdazyl groups might also be suited to participate in ET processes in materials. Thermodynamic information concerning the ET reactions of verdazyls was obtained in a detailed electrochemical study by Gilroy et al. and in an experimental and theoretical study on two verdazyl compounds substituted by electron-rich aromatic donors by Haller et al. [34,35]. Kinetic or structural aspects of ET reactions in verdazyls were, however, not investigated in detail in these studies.

The cited studies of the physical properties of verdazyls demonstrate the need for versatile functionalization patterns to control the properties of the radical compounds. Important steps in the development of verdazyl chemistry have been the introduction of protocols that allow access to diversely substituted tetrazinanones, which can be converted to oxoverdazyls by oxidation [36,37,38]. Recently, Pd catalyzed cross-coupling reactions with oxoverdazyls and with saturated Kuhn verdazyls bearing a phenyl substituent in the C6-position have been reported [39,40]. These coupling reactions allow introducing substituents after the radical center has been synthesized and tolerate a large spectrum of functional groups. In this paper, ET reactions in a 3-*p*-iodophenyl substituted Kuhn verdazyl are studied by crystallography, DFT calculations, and EPR spectroscopy. Furthermore, an example of a successful Sonogashira–Hagihara coupling employing a Kuhn verdazyl without bulky substituents in the C6-position is presented. Finally, magnetic data is presented and discussed for all radicals. These three aspects are of relevance for potential applications of verdazyls in MMM.

## 2. Results

This section starts with the syntheses and the crystal structures of all isolated compounds. Then, the magnetic properties of verdazyls **3·**, **5·** and **6·** as well as the ET characteristics of the redox couple **3·**/**3^+^** are presented. Finally, the experimental results are compared to results of DFT calculations, the detailed data are summarized in the Supplementary Materials.

### 2.1. Syntheses

**Scheme 1:**
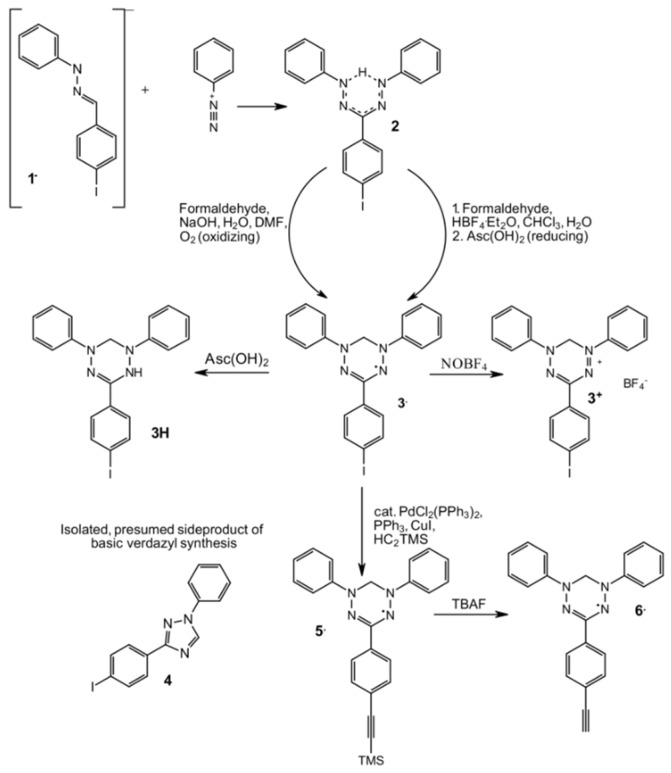
Syntheses conducted in this work. Abbreviations: DMF = dimethylformamide, Asc(OH)_2_ = ascorbic acid, TMS = trimethylsilyl, TBAF = tetrabutylammonium fluoride. For detailed descriptions of the reactions see the Materials and Methods section.

The syntheses of the title compound **3·**, its oxidized and reduced forms **3^+^** and **3H**, its precursor formazan **2** as well as the compounds **5·** and **6·** resulting from the Sonogashira-Hagihara reaction to attach a trimethylsilylacetylene (TMS-acetylene) group with subsequent TMS removal are summarized in Scheme 1, which also includes by-product **4**, obtained during the synthesis of **3·**.

Formazan **2** was synthesized starting from the hydrazone **1** [41], which can be obtained in nearly quantitative yields by refluxing a solution of *p*-iodobenzaldehyde and phenylhydrazine in methanol and subsequent purification by column chromatography. Another, more practical way to obtain **1** is refluxing the same reagents in ethanol instead of methanol. After removing the heat source, **1** precipitates as white, microcrystalline powder, which requires no further purification after washing with cold ethanol. This procedure saves time, but also leads to reduced yields of ~70%. Given the cheap educts of **1**, the second method was preferable in the case at hand. In the next step, **2** was synthesized by slowly adding an aqueous solution of phenyldiazonium chloride to a biphasic, basic solution of **1** (which is deprotonated in-situ to yield **1**), leading to the evolution of an intense red color in the organic phase. After extraction of the organic phase and washing, the raw product was obtained as an oily solid from which pure product could be obtained by trituration and recrystallization. Purification by column chromatography was also possible. Due to the limited solubility of **2** and its intense color, which prevents the experimentalist from seeing whether or not **2** is dissolved completely, it is advisable to divide the raw product into small portions to be purified individually.

Verdazyl **3·** can be obtained by condensing **2** with formaldehyde either under basic conditions in the presence of oxygen in a biphasic medium [34] or under acidic conditions forming **3^+^** initially, with subsequent addition of ascorbic acid Asc(OH)_2_ as reducing agent in a biphasic reaction medium [42]. It appears rather curious that **3·** can be obtained from the same educts under either oxidizing or reducing conditions in one-pot reactions. This implies that the two routes involve strongly different reaction mechanisms and proceed via different intermediate products. Both reaction conditions were tested to see which one performs better in the case at hand. In the basic approach, a DMF solution of **2** was stirred with sodium hydroxide solution and an excess of aqueous formaldehyde. The progress of the reaction was monitored by observing the color change from intense red (color of **2**) to brown (presumably a mixture of **2** and **3·**) to intense green (color of **3·**). Using this approach, **3·** was obtained in a yield of 37% after column chromatography. Interestingly, a quantitative consumption of **2** was not achieved even if the already green solution was stirred for a prolonged time, and small amounts of **2** could be recovered by chromatography. It may be hypothesized, that only one conformation of **2** (in Scheme 1, the red syn-s-cis isomer is given) is reactive, and that the observation of remaining formazan even after long reaction times is related to the occurrence of nonreactive conformations [43,44,45]. In the basic approach to **3·**, triazole **4** was isolated in later fractions of the column. **4** is similar to a byproduct obtained earlier when synthesizing **3Br·**, the bromo analogue of **3·** [46]. Such triazoles have been described as decomposition products of verdazyls formed under much harsher conditions, e.g., heating in benzene to 80 °C for four days or heating to 200 °C [47].

The acidic procedure was slightly modified as compared to the original protocol [42] by using 2–3 times more acid (HBF_4·_Et_2_O), as only then a complete change of color from intense red (color of **2**) to deep purple (color of **3^+^**) was observed. After reduction with ascorbic acid Asc(OH)_2_ under biphasic conditions, **3·** was obtained in a yield of 70% after chromatography. Again, complete conversion of **2** was not achieved. Noteworthy, **3^+^** is reduced twice to yield the leucoverdazyl **3H** if the reduction is conducted without biphasic conditions [42]. This was used to purposefully reduce verdazyl **3·** in methanol containing a large excess of Asc(OH)_2_. The reduction was completed within a few minutes as indicated by the disappearance of the green color of **3·**. Employing an excess of Asc(OH)_2_, the reaction could be conducted without applying laborious Schlenk techniques. As the reoxidation of **3H** by air proceeds sufficiently slow at room temperature, even the purification could be conducted by a standard chromatography procedure, which afforded a slightly green solution of **3H**. This solution can either be used to crystallize **3H** (see below), or to obtain microcrystalline **3H** for potential follow-up reactions by removing the solvent under reduced pressure. Such a convenient method is probably only possible in favorable cases. **3^+^**(BF_4_)^−^ was prepared by adding a solution of (NO)^+^(BF_4_)^−^ in acetonitrile (MeCN) to a dichloromethane (DCM) solution of **3·**, which resulted in an immediate color change from green to purple. The purple solution was left to evaporate, which afforded **3^+^**(BF_4_)^−^ as crystals that resemble metallic copper in terms of color and luster.

Aside from these redox reactions, **3·** was also used as substrate in a Sonogashira–Hagihara coupling reaction with TMSC_2_H followed by removal of the TMS group. At 60 °C, using a catalyst system comprised of CuI, PPh_3_, and Pd(PPh_3_)_2_Cl_2_ and a twofold excess of TMSC_2_H, the reaction was completed in two hours and a yield of 50–55% of **5·** was isolated after chromatography. Interestingly, the green color of the verdazyl disappeared slowly after full consumption of the substrate **3·** and was recovered when washing the solution with water after stopping the reaction. This was taken as indication that the catalyst system reduced **5·** to its leucoform **5H** after completion of the Sonogashira–Hagihara reaction. Concerning this interpretation, it is of relevance to note that it has been shown that verdazyls are readily reduced after forming complexes with Pd(II) [48,49,50]. The quick reoxidation during washing demonstrates that it is not always possible to avoid Schlenk techniques when handling leucoverdazyls as it was the case when preparing **3H**. Noteworthy, no trace of product could be detected by mass spectrometry after a reaction time of 8 h if the Sonogashira–Hagihara reaction was conducted using **3Br·** [46] under otherwise identical conditions. This lower reactivity is in agreement with observations made earlier [39,40].

The TMS group of **5·** was removed with tetrabutylammonium fluoride in THF in the next step. Surprisingly, only 50% yield of **6·** could be obtained after chromatography. The combination of Pd catalyzed cross-coupling and TMS removal was conducted three times with slightly differing conditions (temperature, reaction time, and excess of TMSC_2_H and once under exclusion of oxygen in the deprotection step), and in each case, such low yields in the deprotection step were obtained.

### 2.2. Crystallography

#### 2.2.1. General Remarks and Crystallization Procedures

The crystal structures yield detailed information for all compounds presented herein and selected structural aspects will be discussed below (the crystal structure of **2** and **4** are discussed in the ESI). Such information is especially helpful when rationalizing the magnetic interactions in the solid state structure of verdazyls **3·**, **5·** and **6·**. In the case of **3·**, the crystal data allow elucidating the effect of ET on the structure of verdazyls by comparison with the structures of its redox products **3^+^**(BF_4_)^−^ and **3H**. To exclude the possibility that the observed structures stem from forces exerted by the crystal lattice, the molecular structures of the verdazyls **3·**, **5·** and **6·** as well as for the redox products of **3·** have also been obtained by DFT geometry optimizations. All compounds described in this work crystallized readily if solutions in mixtures of DCM and hexane (a mixture of ether and hexane in the case of **5·**) were left to evaporate, provided the compounds were sufficiently prepurified (e.g., by chromatography). For **3H**, crystallization was conducted in the fridge at 0 °C, to reduce reoxidation to **3·**. In this way, colorless crystals could be obtained, some of which with a slight, green tint. Inspection of the tinted crystals using EPR spectroscopy revealed a narrow signal without resolved hyperfine interactions at a *g* value of ~2.0035, typical for exchange narrowing [51]. The signal showed no dependence on the orientation of the crystal with respect to the magnetic field. These observations are taken as indication that the reoxidized radicals are dispensed either in a microcrystalline form with randomly oriented microcrystals or in an amorphous form on the surface of **3H** rather than as isolated defects in the host lattice, which would presumably lead to highly resolved signals with the typical verdazyl hyperfine splitting. **3^+^**(BF_4_)^−^ was obtained in crystalline form directly from the reaction solution. To improve the crystal size of verdazylium **3^+^**(BF_4_)^−^, the obtained crystals could be redissolved in pure MeCN and then subjected to slow evaporation.

#### 2.2.2. Discussion of the Crystal Structures

**Figure 2:**
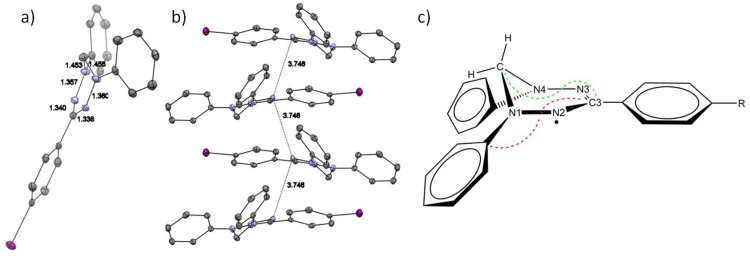
X-ray structure of **3·**. Ellipsoids at 50% probability level. H atoms are omitted for clarity. Color code: grey = carbon, blue = nitrogen, purple = iodine. (**a**) Molecular structure of **3·**. (**b**) Slipped stacking of **3·** along the *c*-axis. The shortest distances between atoms with large fractions of spin density are indicated. (**c**) Numbering scheme used in the discussion of the molecular structures of the verdazyls and their redox products. The red and the green dashed lines indicate one of the two dihedral angles of the CNNC_Ph_ or the CNNCH_2_ moieties, respectively.

Verdazyl **3·** has a structure similar to **3Br·** [52] and is shown in Figure 2a,b. The heterocyclic ring of **3·** is not planar due to the CH_2_ group, which sticks out of the NNCNN plane by ~0.60 Å. Another way of describing the geometry of the CH_2_ group is by the dihedral angles C_3_N_2_N_1_CH_2_ and C_3_N_3_N_4_CH_2_ (Figure 2c), which amount to 21.20° and 23.55°, respectively. The orientation of the phenyl rings with respect to the heterocycle are best described by the C_3_N_2_N_1_C_Ph_ and C_3_N_3_N_4_C_Ph’_ dihedral angles, amounting to 171.35° and 166.05°, respectively. The N-N bonds in **3·** have a length of ~1.36 Å and are elongated in comparison to its precursor **2** by approximately 0.06 Å. This is plausible, as the unpaired electron of verdazyls resides in an antibonding orbital and thereby reduces the bond order of the N-N bonds in **3·** as compared to **2**. In the crystal structure of **3·**, the molecules form linear chains along the *c*-axis, which can be described as a form of highly slipped stacking (Figure 2b) [53]. In these chains, the closest contacts between atoms that carry big fractions of spin density (see the Theory section) are formed between only one nitrogen atom per molecule at a distance of 3.75 Å. One reason preventing the formation of unslipped face-to-face stacks, in which a higher number of close contacts between nitrogen atoms would occur, might be the lack of planarity of the heterocyclic ring in **3·**.

**Figure 3:**
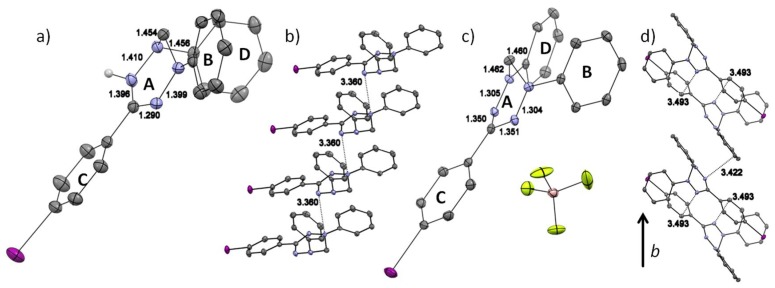
X-ray structures **3H** and **3^+^**[BF_4_]^−^. Ellipsoids at 50% probability level. H atoms are omitted for clarity. Color code: grey = carbon, blue = nitrogen, purple = iodine, rose = boron, yellow = fluorine. (**a**) Molecular structure of **3H**, including the leucoverdazyl H in white. (**b**) Stacking of **3H** along the *a*-axis. (**c**) Molecular structure of **3^+^**(BF_4_)^−^. (**d**) Chains of **3^+^** along the *b*-axis. (BF_4_)^−^ ions are omitted.

Figure 3 shows the crystal structures of the redox products of **3·**. The changes in the molecular structure of **3·** upon reduction or oxidation are important for ET reactions, as these changes contribute to the reorganization energy which determines the rate of the ET [54]. The reduction product **3H** is shown in Figure 3a. **3H** shows an elongation of most bonds within the heterocycle, with the exception of the C-N_divalent_ bond, which is shortened, and the N-CH_2_ bonds, which remain essentially unchanged. These observations are plausible: The N-CH_2_ bonds have single bond character in both **3H** and its precursor **3·**, therefore no change is expected. In contrast, the other bonds change their bond order upon reduction to **3H** and have now either predominant double bond character (C3-N_divalent_) or single bond character (the remaining bonds within the heterocycle) as compared to the situation of delocalized double bonds with intermediate lengths in **3·**. The most notable change however is the orientation of the N1-phenyl group (labeled D in Figure 3a), which bends strongly away from the heterocyclic plane upon reduction to **3H** (dihedral angle C_3_N_H_NC_Ph_ of only 95.7°). Large amplitude molecular motions like this are of interest in molecular machines [55]. Although **3H** is much less planar than **3·**, effective stacking along the *a*-axis is observed, with shorter intermolecular N-N contacts than in **3·** (Figure 3b).

The product of oxidation using NO(BF_4_) **3^+^**[BF_4_]^−^ is depicted in Figure 3c. In **3^+^**, the N-N bonds are significantly shortened as compared to **3·**, in agreement with the removal of one electron from an antibonding orbital. The N-N bond lengths are now very similar to those observed in formazan **2**, which is also true for the C3N2 and C3N3 bond lengths. The two dihedral angles CNNC_Ph_ change to slightly lower values of 158.4° and 162.4° in **3^+^** as compared to **3·**. However, the overall change of the molecular shape upon oxidation is much less drastic as compared to the changes observed upon reduction. This means that ET from verdazyl radicals to verdazylium cations could possibly occur even in the solid state, provided suitable packing in the crystal structure occurs. In the case at hand, the cations **3^+^** form chains of head-to-tail dimers (rings A and C face each other) along the *b*-axis, despite the lack of planarity of the heterocyclic ring (Figure 3d). This arrangement places the HOMO and LUMO orbitals of the two molecules comprising a dimer in direct vicinity (see ESI for the HOMO and LUMO orbitals of **3^+^**). The planes of the rings are oriented approximately perpendicular to the axis of the molecular chains and point along $\left(10\bar{1}\right)$. ET events within a dimer would probably occur along this axis. Long range electron migration would require movement along a suited pathway through the crystal, however, which is not observed in the structure of **3^+^**[BF_4_]^−^. In the context of conductivity, it would be interesting to obtain co-crystals of **3·** and **3^+^**[BF_4_]^−^. Unfortunately, using the same crystallization conditions as for the pure compounds but with mixed solutions of **3·** and **3^+^**[BF_4_]^−^ did not yield co-crystals. Instead, only crystals which had either the unit cell parameters of **3·** or of **3^+^**[BF_4_]^−^ were obtained. Analogously, the same result was obtained when repeating the co-crystallization experiment with **3H** and **3·**.

**Figure 4:**
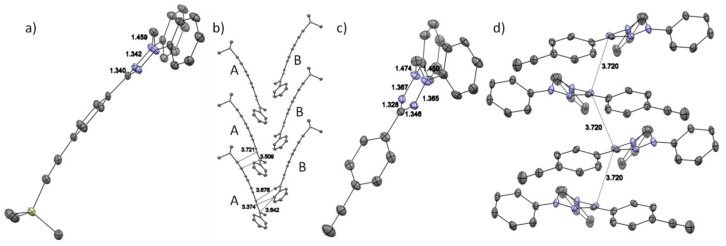
X-ray structures of **5·** and **6·**. Ellipsoids at 50% probability level. H atoms are omitted for clarity. Color code: grey = carbon, blue = nitrogen, beige = silicon. (**a**) Molecular structure of **5·**. (**b**) Fishbone pattern of **5·** along the *c*-axis. (**c**) Molecular structure of **6·**. (**d**) Slipped stacking of **6·** along the *c*-axis.

Finally, the structures of **5·** and **6·** are discussed (Figure 4). The molecular structure of **5·** is shown in Figure 4a and shows similar bond lengths in the heterocyclic ring as **3·**. The molecular structure of **5·** in its crystalline state has a mirror plane dissecting the heterocycle. The dihedral angles C_3_NNCH_2_ and C_3_NNC_Ph_ are similar to those in **3·** and amount to 20.97° and 171.14°, respectively. The TMS-ethinylene unit deviates by about 8–9° from collinearity, similar to values observed before in molecules with phenylene–ethinylene backbones [56,57,58,59]. The packing of **5·** strongly differs from the packing of **3·**, probably due to the bulky TMS-C_2_ substituent, and the molecules are arranged in a fishbone pattern along the *c*-axis (Figure 4b). As can be seen in Figure 4b, the fishbone pattern can be interpreted as a linear chain in which the molecules are arranged in an alternating ABAB fashion, where molecules A and B belong to the different sides of the fishbone pattern. The CH_2_ group as well as the N-phenyl rings of molecules B point towards the heterocyclic plane of molecule A and vice versa. Within one side of the fishbone pattern, two molecules are stacked with the C_2_ unit of one molecule being above the middle of the heterocyclic plane of the other molecule where the node of the SOMO is located. These contacts do not involve atoms that carry large fractions of spin density and are indicated in Figure 4b.

The structure of compound **6·** is depicted in Figure 4c. The heterocyclic moiety is similar to the other verdazyls presented above. The crystal structure is isomorphic to the structure of **3·**, and consequently the same stacking pattern along the *c*-axis is observed with the shortest intermolecular N-N distance amounting to 3.72 Å (Figure 4d).

### 2.3. EPR Spectroscopy

**Figure 5:**
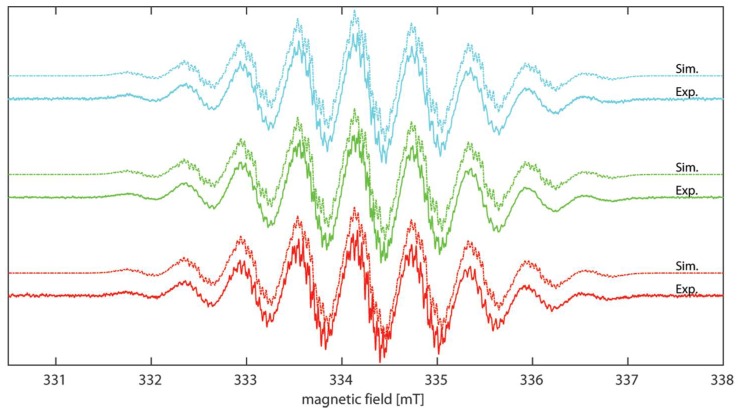
EPR spectra of degassed, deuterated DCM solutions of **3·** (red), **5·** (green), and **6·** (cyan) and simulations.

Figure 5 shows the EPR spectra of radicals **3·**, **5·** and **6·** along with their simulations. The EPR parameters are given in Table 1 and are similar to what has been observed previously in **3Br·** [46]. Notably, the isotropic hyperfine coupling constants *A_iso_* used for the protons are qualitatively in agreement with the spin density distribution in verdazyls (see the Theory section).

**Figure 6:**
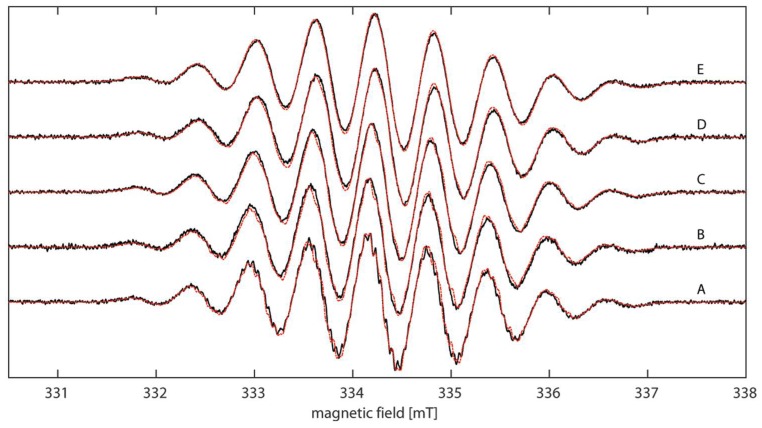
EPR spectra of degassed, deuterated MeCN solutions of **3·** mixed with different concentrations of **3^+^**[BF_4_]^−^. Black, full lines: experimental spectra. Red, dashed lines: simulations. Samples A, B, C, D, and E contain concentrations of 0, 2.5, 5, 10, and 20 µM of **3^+^**[BF_4_]^−^,respectively.

The Lorentzian linewidth parameter given in Table 1 can be used to study electron self-exchange reactions of verdazyls. To that end, solutions of 3. (fixed concentration of 1 mM) have been mixed with solutions of 3^+^(BF4)—(variable concentrations up to 20 mM) and the effect on the linewidth of the EPR signals was monitored by cw EPR spectroscopy (Figure 6). The reason for the line broadening in such mixtures are collisions between radical molecules 3. and cations 3^+^, which lead to ET reactions according to Equation (1):

(1) $$3\cdot +3^{+}\to 3^{+}+3\cdot \;$$

Such ET events limit the lifetime of radical **3·** and therefore contribute to the Lorentzian linewidth of the EPR absorption line. The change in linewidth is proportional to the rate constant $k_{obs}$ of the ET and can be described by Equation (2) [61]:

(2) $$\;\Delta lwpp=\frac{\left(1-p_{i}\right)}{\pi \sqrt{3}\gamma_{e}}k_{obs}\left[3^{+}\right]\;$$

Here, Δlwpp is the change of the peak-to-peak linewidth (Lorentzian contribution), $p_{i}$ is the population factor of the i’th EPR line and $\gamma_{e}$ is the gyromagnetic ratio of the unpaired electron amounting to 28.03 MHz/mT. Simulations of the spectra with different linewidth parameters as a function of [**3^+^**] together with Equation (2) yield $k_{obs}=6.8\times 10^{8\;}M^{-1}s^{-1}$, with an estimated error of about ±20%. Taking into account a correction for diffusion using Equation (3) [61]

(3) $$\;\frac{1}{k_{ET}}=\frac{1}{k_{obs}}-\frac{1}{2k_{diff}}\;$$

with a diffusion rate constant $k_{diff}$ depending on the solvent viscosity η as described by Equation (4)

(4) $$\;k_{diff}=\frac{8RT}{3\eta }\;$$

leads to a rate constant of $k_{ET}=6.9\times 10^{8}\;M^{-1}s^{-1}$ for the ET, which differs marginally from $k_{obs}$. The values for $k_{obs}$ and $k_{ET}$ reproduce the linewidth alteration of the central and the outmost line equally well and are about 2.7 times higher than the value reported for TEMPO/TEMPO^+^ redox couple in the same solvent, indicating that electron self-exchange is less hindered in the redox couple **3·**/**3^+^** [61]. This increased rate will be taken up and discussed further in the Theory section.

### 2.4. Magnetometry and Conductivity

Verdazyls **3·**, **5·** and **6·** have been subjected to solid state magnetometry. In this section, the experimental data are discussed based on structural arguments, whereas a comparison with the results of broken symmetry (BS) DFT calculations is presented in the Theory section. The magnetic interactions can be quantified using the exchange coupling constant *J* used in the exchange spin-Hamiltonian *H_exc_* (Equation (5)) [62]:

(5) $$\;H_{exc}=-JS_{1}S_{2}\;$$

**Figure 7:**
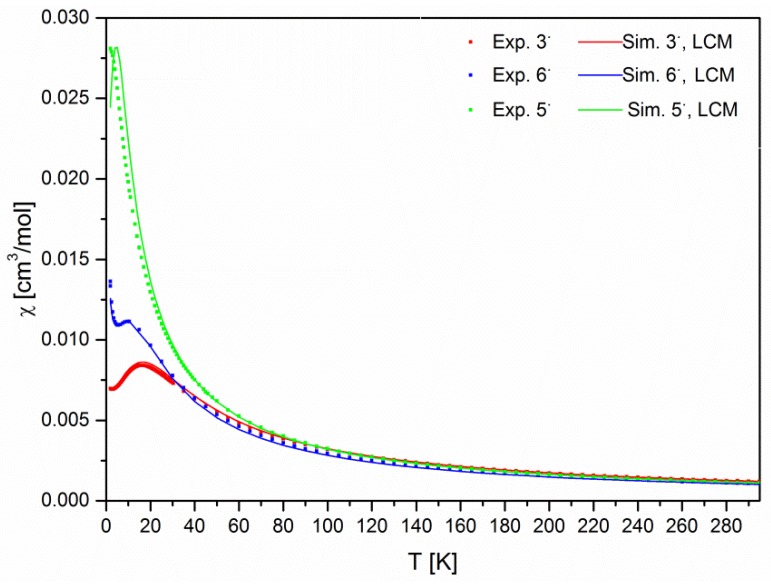
Experimental susceptibility *χ* (dotted lines) and simulations (full lines) using the LCM.

A negative value of *J* signifies AF interactions between the spins *S_i_* (i = 1 or 2 in a radical pair) in Equation (5). Figure 7 shows the magnetic susceptibility *χ* as a function of the temperature *T* along with simulations for all three compounds. For all compounds, the magnetic susceptibility approaches finite values at low temperatures, indicating AF interactions. This is also apparent from the plots of *χT* vs. *T*, which show a steep decrease at temperatures below 50 K (see ESI). In the case of **3·**, the molecules are arranged in linear chains with close intermolecular contacts formed by the spin bearing nitrogen atoms (see above). Thus, the SOMOs of the compounds are expected to overlap strongly, which generally leads to AF coupling [24,62]. The obtained data for **3·** was fitted by using the linear chain model (LCM) for *χ* [62], in accordance with the observed packing motive and yielding an exchange coupling constant of *J*(**3·**) = −18 cm^−1^. To account for the increase of the magnetic susceptibility at temperatures below ~5 K, a contribution of 0.4% of uncoupled molecules has been introduced into the simulation. Such contributions may arise as a consequence of defects in the crystal structure. The susceptibility data of **6·** can be analyzed in the same way as the data for **3·**, since their crystal structures are isomorphic. Using the LCM, *J*(**6·**) = −12 cm^−1^ is obtained, with a paramagnetic contribution amounting to 2.4%. More cumbersome than the paramagnetic contribution is the fact that the susceptibility values in the case of **6·** are consistently lower than theory would predict by about 17% (this was taken into account by using an empirical correction factor in the simulations). This observation is especially puzzling as the material was highly crystalline and the elemental analysis also indicated a high degree of purity. Considering these two analytical results, only the reduced leucoform of **6·** or the HC_2_-substituted analogue of formazan **2**, which could possibly form during synthesis of **6·**, appear to be suited candidates for diamagnetic contaminations. However, neither of these two candidates could be evidenced by IR spectroscopy (leucoform of **6·**) or TLC (HC_2_ substituted formazan), which should have been facile if the contaminations made up ~17% of the material.

The most complicated case of magnetic coupling is encountered in **5·**. As discussed above, no stacking interactions that involve atoms with large spin density on both interacting molecules are observed. Based on that simple argument, very weak interactions are expected. This is also found experimentally, with **5·** being the only example in which the susceptibility does not go through a maximum and resembles the curve of an uncoupled paramagnetic sample until very low temperatures. In the crystal structure of **5·**, each molecule interacts with two types of partner molecules A and B (see Figure 4). Thus, the Majumdar–Ghosh chain model might be appropriate here [63,64], which may give rise to phenomena like spin frustration [65,66,67]. However, theoretical data obtained by the BS-DFT methodology suggests that spin frustration is not expected here and that using the LCM with AF interactions might be a suited approximation for **5·** (see the Theory section for more details). Although no fully satisfying simulation was obtained with this approach, the simulation allowed estimating effective AF interactions 0 > *J_eff_*(**5·**) > −7.5 cm^−1^. A similar estimate was obtained using the Curie–Weiss law (simulation not shown). As with **6·**, the magnetic susceptibilities of **5·** are consistently too low by ~10% (see also the plot *χT* vs. *T* in the ESI), despite good crystallinity of the sample and an elemental analysis consistent with a highly pure material.

**Figure 8:**
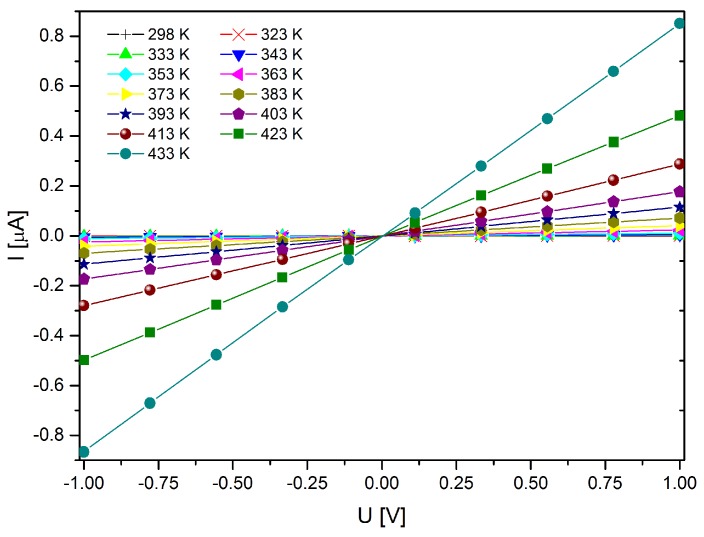
Current *I* as a function of voltage *U* for **3^+^**[BF_4_]^−^ at different temperatures.

Encouraged by the metallic appearance of **3^+^**[BF_4_]^−^ and the rapid ET reactions in **3·**/**3^+^**, conductivity measurements have been conducted on crystalline **3^+^**[BF_4_]^−^. It was indeed possible to obtain linear plots of the current *I* as a function of the voltage *U*, but the observed conductivities were low. Figure 8 shows such plots for one sample at different temperatures. The increasing conductivity at high temperatures is typical for semiconductors. Using Equation (6), which describes the conductivity *σ* as a function of the temperature [68], allows obtaining the band gap *∆E* by plotting negative logarithmic reciprocal resistances *−ln(1/R)* as a function of the reciprocal measurement temperature *1/T.*

(6) $$\;\sigma =\frac{1}{R}=\sigma_{0}e^{-\frac{\Delta E}{2kT}}\;$$

The corresponding plots obtained on three different samples are shown in the ESI and yield ∆*E* = 2.13 ± 0.41 eV for the band gap of **3^+^**[BF_4_]^−^, comparable to the value of GaP [68]. As an additional control, conductivity measurements were also conducted on **3·**. There, a bandgap of 3.92 eV was obtained, which is characteristic for an insulator. A reason for the rather low conductivity of **3^+^**[BF_4_]^−^ might be the dimeric structure of the cations **3^+^** in the solid state, as opposed to an extended stacking structure which could provide better overlap between the relevant orbitals and thus a better pathway for long range charge transport (see also the ESI) [69].

### 2.5. DFT Calculations

**Figure 9:**
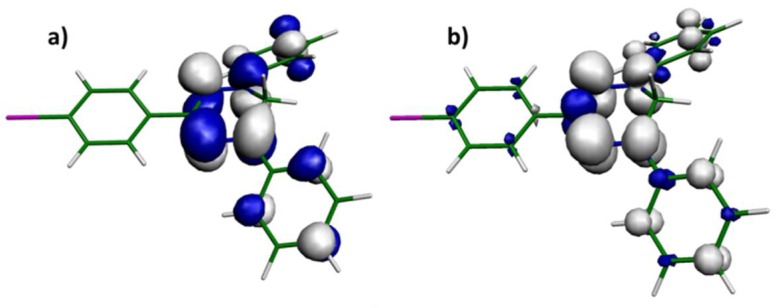
SOMO (**a**) and spin density (**b**) of **3·**. White lobes indicate positive sign in the spin density.

DFT calculations have been performed with three goals in mind: (a) Obtaining the molecular structures of the verdazyls **3·**, **5·** and **6·** and following the structural changes occurring during redox reactions in **3·**, (b) rationalizing the rate of ET processes in the redox couple **3·**/**3^+^,** and (c) getting further confirmation of the magnitude of the magnetic interactions in the solid state structures of verdazyls **3·**, **5·** and **6·**. Concerning the first goal, DFT geometry optimizations afforded molecular structures that are nearly indistinguishable from the experimental crystallographic structures in all cases. Table 2 summarizes relevant structural parameters obtained experimentally and by DFT. The DFT structures of the supposedly symmetric molecules **3·**, **3^+^** and **6·** are much more symmetric than their crystallographic structures with respect to the heterocyclic bond lengths and the dihedral angles C_3_N_i_N_j_CH_2_ and C_3_N_i_N_j_C_Ph_ (i and j = 2 and 1 or 3 and 4 for the two possible dihedrals). Aside from the geometries, DFT calculations also afford orbital and spin density plots, an example given in Figure 9 (further examples in the ESI).

Using the results of the DFT geometry optimizations, it is possible to calculate the inner-sphere reorganization energy *λ_i_*, which is determined by the structural changes within the molecule partaking in the electron self-exchange. *λ_i_* contributes together with the outer-sphere reorganization energy of the molecule’s solvent cage *λ_o_* to the total reorganization energy *λ* (Equation (7)):

(7) $$\lambda =\lambda_{i}+\lambda_{o}.\;$$

Using Marcus’ theory, exponential dependence of *k_ET_* on *λ* is expected (Equation (8)) [54]:

(8) $$\;k_{ET}=\alpha e^{-\frac{\lambda }{4RT}}\;$$

Equation (8) allows relating the electron self-exchange rate to the structural changes observed upon oxidation of **3·** and comparing the obtained results to the TEMPO/TEMPO^+^ redox couple studied before [61] (the factor *α* is treated as an empirical proportionality constant). To compute $\lambda_{i}$ according to Nelsen et al. [70], the single point energy of **3·** is calculated in the optimized cation geometry of **3^+^** and vice-versa. This yields the reorganization energies for the radical **3·** and the cation **3^+^**, the sum of the two energies being equal to the total inner-sphere reorganization energy *λ_i_*. For **3·**/**3^+^**, this yields *λ_i_*(**3·**/**3^+^**) = 55.4 kJ/mol, whereas a value of 97.9 kJ/mol is obtained for the TEMPO/TEMPO^+^ redox couple. Thus, according to Equation (8) and accounting only for *λ_i_*, an increase in *k_ET_* by a factor of ~73 would be expected when going from TEMPO/TEMPO^+^ to **3·**/**3^+^**. Even if the same tunneling correction as used by Grampp and Rasmussen is used, an increase by a factor of ~8.5 would be expected [61]. Both values exceed the experimentally observed increase by a factor of 2.7 significantly. One possible explanation for this is the larger size of **3·** as compared to TEMPO, which might lead to an increased outer-sphere reorganization energy *λ_o_*, thereby counterbalancing the lower value of *λ_i_*.

**Figure 10:**
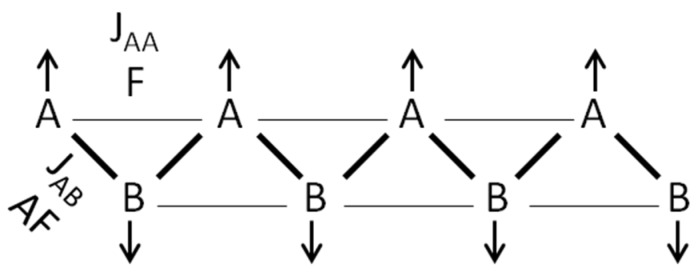
Schematic representation of the Majumdar–Ghosh chain model for the antiferromagnetic-ferromagnetic (AF-F) case [66].

Finally, the exchange interactions observed by magnetometry are compared to the results of BS-DFT calculations, which have been proven to be a useful tool to obtain exchange coupling constants *J* [71,72,73]. First, the LCM compounds **3·** and **6·** are discussed. For these compounds, BS-DFT calculations predict AF exchange coupling constants between two stacked molecules amounting to *J*(**3·**,BS-DFT) = −13.34 cm^−1^ and *J*(**6·**, BS-DFT) = −7.52 cm^−1^. For both compounds, the sign of the interaction is predicted correctly and also the ratio *J*(**3·**)/*J*(**6·**) of the theoretical values is similar to the experimental ratio. The absolute, experimental values are larger by a factor of ~1.45 than the theoretical ones, which can still be considered good agreement between theory and experiment. In the more complicated case of **5·**, two dimer interactions had to be investigated by BS-DFT to account for the ABAB fishbone pattern stacking. For the two dimers, exchange coupling constants of *J*(**5·**, AB, BS-DFT) = −21.10 cm^−1^ and *J*(**5·**, AA, BS-DFT) = +0.74 cm^−1^ were obtained. Noteworthy, these coupling constants indicate the simultaneous occurrence of AF and ferromagnetic interactions, but the ferromagnetic coupling constant is very low. If the existence of this very weak ferromagnetic coupling path is accepted, a Majumdar–Ghosh chain without spin frustration is expected (Figure 10). Such chains behave very similar to purely AF coupled chains, but with effectively increased AF interactions (i.e., maxima of *χ* occurring at higher temperatures than in the LCM case with the same value for the AF coupling constant) [66]. Given the low value of *J* (**5·**, AA, BS-DFT), an equally valid interpretation may be the complete neglect of this coupling constant. In this case, the resulting magnetic structure would be described by an AF LCM. For both cases, the LCM should thus yield qualitatively correct results. The quantitative agreement between DFT and experiment is worse for **5·** than in the other two cases. The reason for this is tentatively assigned to an overestimation of the delocalization of the spin density on the phenyl rings, which are in close contact with the heterocyclic plane of the adjacent molecule in the AB dimer [74,75,76,77,78]. Noteworthy, the delocalization error was linked to the implementation of the Hartree–Fock exchange (HFX) interaction in the employed theory [74], which was also found to affect the accuracy of the prediction of *J* values in coupled radical pairs [79,80]. Thus, improvement of the calculated values could possibly be achieved by optimizing the amount of HFX in the functional. However, since this optimization is challenging, and given the already good agreement between theory and experiment for the above examples, such an optimization was not attempted in this study.

## 3. Materials and Methods

### 3.1. Syntheses

General procedures

Commercially available chemicals and solvents were purchased from Sigma-Aldrich (Saint Louis, MO, USA) and used without further purification. Silica column chromatography was performed using E. Merck silica gel (60 Å pore size, 40–63 µm particle size, 230–400 mesh). Aluminum oxide column chromatography was performed using neutral aluminum oxide with a water content of 5%. IR and UV/Vis spectra have been recorded and are shown in the ESI.

*N*-Phenyl-p-iodobenzaldehyde-hydrazone **1**

1.5 g phenylhydrazine (13.89 mmol, 1.37 mL) were added to a solution of 3.29 g *p*-iodobenzaldehyde (14.18 mmol) in EtOH. The yellow solution was refluxed for 30 min before removing the heat source, which led to the occurrence of a slightly yellow, microcrystalline precipitate. After cooling down to room temperature, the precipitate was washed with cold ethanol, yielding **1** without requiring further purification (3.13 g, 70%).

EI-MS: *m*/*z* 322.0 (100; [C_13_H_10_N_2_I]^+^, [M]^+^).

1,5-Diphenyl-3-p-iodophenyl-formazan **2**

4.06 g sodium carbonate (38.29 mmol) were dissolved in water (15 mL) and mixed with a solution of 3 g of **1** (9.317 mmol) and 0.31 g tetrabutylammonium bromide (0.96 mmol) in DCM (80 mL). Meanwhile, aniline (0.83 mL; 9.089 mmol), hydrochloric acid (2.5 mL, 37%), and water (5 mL) were stirred in a round-bottom flask at 0 °C while slowly adding a solution of sodium nitrite (0.786 g, 11.384 mmol) in water (10 mL) to yield a solution of phenyldiazonium salt. This solution was stirred for 10 min and then added slowly to the first solution over the course of 10 min. Stirring the resulting mixture at 0 °C for 20 min, then at room temperature for another 40 min, yielded a dark red solution. For purification, the product was extracted with DCM, washed with water twice and dried over sodium sulfate, then taken to dryness in vacuum to yield an oily, deeply red solid. The raw product was purified by trituration with methanol with subsequent recrystallization or by chromatography (stationary phase AlOx, eluent cyclohexane/DCM 4:1) followed by recrystallization (2.94 g, 76%).

ESI-MS: *m/z* 425.026 (100; [C_19_H_14_N_4_I]^+^, [M − H]^+^). Elemental Analysis: Calcd.: C, 53.53; H, 3.55; N, 13.15. Found: C, 53.84; H, 3.58; N, 12.78.

1,5-Diphenyl-3-p-iodophenyl-verdazyl **3·**

Basic procedure: 1.604 g of **2** (3.763 mmol) were dissolved in DMF (64 mL) and mixed with 35% formaldehyde solution (4.2 mL; 53.41 mmol) and 2 M sodium hydroxide solution (8.7 mL). Stirring at room temperature for 5 h gave a dark green solution that was then washed with diethyl ether and brine. The organic phase was dried over sodium sulfate and filtered. Chromatography (SiO_2_; cyclohexane/DCM 3:1) yielded **3·** as a green solid after removal of the eluent (0.608 g, 37%). Crystals suitable for X-ray crystallography were grown by evaporation of a DCM/*n*-hexane solution.

Acidic procedure: 2.001 g of **2** (4.694 mmol) was dissolved in chloroform (120 mL) and water (23 mL) was added. An ethereal solution of fluoroboric acid (55%, 18.3 mL) was added in three portions and a color change from red to purple was observed. The solution was stirred for 3 h at 60 °C. After cooling down the mixture, aqueous ammonia (25%) was added until the pH was basic. Then, the purple solution was treated with L-ascorbic acid (4.148 g; 23.552 mmol) in water (120 mL) leading to a change of color to green in the course of 15 min. For purification, the green solution was washed with water three times and extracted with chloroform, then dried over sodium sulfate. Column chromatography (SiO_2_, cyclohexane/DCM 1:1) yielded **3·** as a green solid.

ESI-MS: *m/z* 439.042 (100; [C_20_H_16_N_4_I]^+^, [M]^+^). Elemental Analysis: Calcd.: C, 54.68; H, 3.67; N, 12.76. Found: C, 54.55; H, 3.72; N, 12.43.

Since **4** was isolated after basic synthesis of **3·**, we also give the MS analytics of **4** here: ESI-MS: *m*/*z* 348.000 (100; [C_14_H_11_N_4_I]^+^, [M + H]^+^).

1,5-Diphenyl-3-p-iodophenyl-verdazylium tetrafluoro borate **3^+^**(BF_4_)^−^

A solution of 25 mg (0.057 mmol) of **3·** in 4 mL of DCM was mixed with a solution of 4.25 mg (0.071 mmol) of (NO)^+^(BF_4_)^−^ and stirred at room temperature, leading to an immediate color change from green to purple. After stirring for one minute, the solution was left to evaporate, yielding crystals that were suited for X-ray structure determination and resembled copper in their appearance (30 mg, 85%).

ESI-MS: *m*/*z* 439.042 (100; [C_20_H_16_N_4_I]^+^, [M]^+^). Elemental Analysis: Calcd.: C, 45.57; H, 3.06; N, 10.63. Found: C, 45.80; H, 3.52; N, 10.14.

1,5-Diphenyl-3-p-iodophenyl-leucoverdazyl **3H**

54 mg (0.123 mmol) of **3·** were dissolved in 3 mL of DCM and mixed with 3 mL MeOH and 24 mg (0.136 mmol) of ascorbic acid. The solution was stirred at room temperature, upon which the green color of the solution vanished in the course of minutes and was replaced by a slightly yellow color. Then, the solvents were removed under reduced pressure. DCM was added to the resulting dirty yellow solid, thereby redissolving **3H** but not remaining ascorbic acid or oxidized ascorbic acid. The solution was then filtered using a SiO_2_ column and the resulting slightly green DCM solution was mixed with *n*-hexane and left in the fridge (~4 °C), affording colorless crystals suitable for X-ray crystallography. (17 mg, 32%).

ESI-MS: *m/z* 441.058 (100; [C_20_H_18_N_4_I]^+^, [M + H]^+^), 439.042 (100; [C_20_H_16_N_4_I]^+^, [M − H]^+^). Elemental Analysis: Calcd.: C, 54.55; H, 3.89; N, 12.73. Found: C, 54.52; H, 3.86; N, 12.75.

1,5-Diphenyl-3-p-((trimethylsilyl)ethinyl)phenyl-verdazyl **5·**

140 mg (0.319 mmol) of **4·** were dissolved in 15 mL of TEA and 2.5 mL of DMF at 60 °C under Schlenk conditions. 6 mg (0.030 mmol) of CuI, 17 mg (0.061 mmol) of PPh_3_, and 14 mg (0.020 mmol) Pd(PPh_3_)_2_Cl_2_ as well as 0.12 mL (~84 mg, 0.857 mmol) ethynyltrimethylsilane were added to the green solution. The reaction was stopped after 2 h at 60 °C and the solution was washed with water and then dried over Na_2_SO_4_ before removal of the solvents at the rotary evaporator. The resulting green oil was subjected to column chromatography using SiO_2_ and a mixture of cyclohexane and ether (1.5:1) as eluent. The green fractions were collected and the solvents were removed under reduced pressure to obtain **5·** as green solid (68 mg, 52%). A portion of the product was redissolved in ether and *n*-hexane to afford single crystals suitable for X-ray crystallography after evaporation.

ESI-MS: *m*/*z* 409.184 (100; [C_25_H_25_N_4_Si]^+^, [M]^+^). Elemental Analysis: Calcd.: C, 73.36; H, 6.16; N, 13.69. Found: C, 73.24; H, 6.31; N, 13.49.

1,5-Diphenyl-3-p-ethinylphenyl-verdazyl **6·**

60 mg (0.146 mmol) of **5·** were dissolved in 5 mL of THF and mixed with a solution of 63 mg (0.195 mmol) of tetrabutyl ammonium fluoride trihydrate in 15 mL of THF yielding a brown solution which was stirred for 3 h at room temperature. Then, the solution was washed with water and dried over Na_2_SO_4_ before removing the solvent under reduced pressure. The raw product was redissolved in a mixture of DCM and cyclohexane (1:1) and passed through a filter column (SiO_2_). Removal of the solvent afforded a green, solid product (24 mg, 48%). Crystals suitable for X-ray crystallography were grown by slow evaporation of a DCM/*n*-hexane solution.

ESI-MS: *m*/*z* 337.145.184 (100; [C_22_H_17_N_4_]^+^, [M]^+^). Elemental Analysis: Calcd.: C, 78.31; H, 5.08; N, 16.61. Found: C, 78.06; H, 5.19; N, 16.25.

### 3.2. Magnetometry and Conductivity Measurements

Magnetometric measurements have been performed using a vibrating sample magnetometer in the PPMS device (QuantumDesign, San Diego, CA, USA) at field strengths of 1 T in a temperature range of 1.9–298 K. Diamagnetic corrections for the sample tube as well as for the sample itself have been applied [62]. Conductivity measurements were conducted on a Keithley Source Measure Unit. Three independent crystal samples of **3^+^**[BF_4_]^−^ have been used for measuring the DC *I* vs. *U* curve, starting at room temperature and increasing the measurement temperature stepwisely up to a temperature of 433 K.

### 3.3. DFT Calculations

DFT geometry optimizations and single point energy evaluations have been performed using the Kohn–Sham and the unrestricted Kohn–Sham formalism for closed and open shell molecules, respectively, employing the B3LYP functional, a def2-tzvp basis set, and D3 dispersion correction as implemented in the ORCA program package [78,81,82,83,84,85]. The exchange coupling constants *J* observed in the solid state structures of **3·**, **5·** and **6·** were calculated using the broken symmetry DFT [86] approach and Equation (9).

(9) $$\;J=-\frac{2\left(E_{HS}-E_{BS}\right)}{{\langle S^{2}\rangle }_{HS}-{\langle S^{2}\rangle }_{BS}}\;$$

For the BS-DFT calculations, dimers from the crystal structures of the respective molecules have been taken as input geometry without further optimization steps. Spin contamination was found to be low with ${\langle S^{2}\rangle }_{HS}\;<\;2.058$ in all presented examples. Additionally, ${\langle S^{2}\rangle }_{BS}$ values close to 1 were obtained, indicating strong localization of the *α* and *β* spins on just one molecule, in agreement with the sum over the spin populations of the two molecules in each dimer amounting to nearly ±1 on the respective monomer [87].

### 3.4. EPR Sample Preparation

To obtain the EPR spectra shown in Figure 5, solutions of **3·**, **5·** and **6·** in deuterated DCM have been prepared in an argon atmosphere at a concentration of 150 µM. For measurements of the electron self-exchange rate, solutions in deuterated MeCN with concentrations of 1 mM for **3·** and 0, 2.5, 5, 10, and 20 mM for 3^+^[BF_4_]^−^ (A, B, C, D, and E in Figure 6, respectively) have been prepared in an argon atmosphere. The solvents were degassed using repeated freeze-pump-thaw cycles prior to use. The sample solutions were filled into 1-mm-diameter EPR quartz tubes, which were slid into gas-tight Young tubes.

### 3.5. EPR Measurements

All cw EPR experiments were conducted at X-band frequency on a Bruker EMXmicro (Bruker, Billerica, MA, USA) EPR spectrometer with the EMX standard resonator (4119HS).

### 3.6. X-ray Diffractometry

The crystallographic studies have been performed on a Bruker D8-venture diffractometer (**2**, **3**, **5·**, and **6·**), a Bruker X8-KappaApexII (**4** and **2**), and a STOE-IPDS-2T diffractometer (**3H**). The diffractometers were equipped with a low-temperature device (Oxford Cryostream 700er series, Oxford Cryosystems, Oxford, UK); 123(2)K for **3H**; Oxford Cryostream 800er series (Oxford Cryosystems); 100(2)K for **2**, **3**, **5·**, **6·**), and Bruker Kryoflex I, Bruker (AXS); 100(2)K for **2**, **4**). The data collections were done by fine-slicing ϕ and ϖ-scans and corrected for background, polarization and Lorentz effects. An empirical absorption correction was applied for all data sets. The structures were solved by the intrinsic phasing procedure implemented in ShelxT [88] and refined anisotropically by the least-squares procedure implemented in ShelxL [89]. Hydrogen atoms were included isotropically using the riding model on the bound carbon atoms, except for **3H**, where the N4-bonded H atom was located in the difference density map.

The crystals of **4** turned out to be merohedrally twinned (twin law: −1 0 0 0 −1 0 1 0 1). The twin-refinement lead to a batch scale factor of 38.7(4)% for the minor twin component. CCDC-1851443 to CCDC-1851449 contain the supplementary crystallographic data for this paper, which can be obtained free of charge from The Cambridge Crystallographic Data Centre via www.ccdc.cam.ac.uk/data_request/cif.

## 4. Conclusions

In this work, the suitability of Kuhn verdazyls as building blocks in MMMs was investigated in an exemplary study on 1,5-diphenyl-3-*p*-iodophenyl-verdazyl and its follow-up redox and cross-coupling products, focusing on the magnetic and electronic properties. The investigation of the physical properties was backed up by detailed structural analyses using crystallography supported by DFT structure optimizations. With respect to chemical properties, it was demonstrated that it is possible to use a Kuhn verdazyl carrying only H-atoms on C6 as substrate in Sonogashira–Hagihara cross-coupling reactions, which was previously only reported for oxo-verdazyls [39] or verdazyls carrying a bulky phenyl substituent on C6 [40]. In this way, the three different Kuhn verdazyls **3·**, **5·** and **6·** could be obtained, crystallized, and subjected to magnetometric measurements. All compounds showed weak AF coupling. The signs and also the magnitudes of the exchange coupling constants could be reproduced by BS-DFT calculations, although the theoretical *|J|* value for **5·** was somewhat overestimated, possibly owed to an overestimation of the spin density delocalization on the *N*-phenyl substituents. Qualitatively, the low values for |*J|* are probably caused by the lack of spatial proximity and coplanar face-to-face stacking of the heterocyclic planes of the Kuhn verdazyls, with strongly slipped stacks of the heterocyclic planes in **3·** as well as in **6·** and no stacking of the heterocyclic planes at all in **5·**. The tendency to avoid coplanar face-to-face stacking was also noticed in earlier examples [15]. It could be reasoned, that the tendency to avoid face-to-face stacking with other verdazyls or any planar moiety in general observed for Kuhn verdazyls is caused by the nonplanar structure of the heterocycle. However, the even less planar leucoverdazyl **3H** shows a structure with much shorter and also more intermolecular N-N contacts as compared to the parent radical **3·**. Furthermore, the cations in the oxidation product **3^+^**(BF_4_)^−^ form stacked head-to-tail dimers despite being structurally practically identical to the parent radical compound. These observations imply that steric reasons alone are insufficient to explain the stacking behavior of Kuhn verdazyls. Given the obtained results, oxoverdazyls (as opposed to Kuhn verdazyls) remain the better candidate to achieve comparatively strong magnetic coupling in purely organic, verdazyl based materials. In addition to the magnetic properties, the behavior of **3·** in ET reactions was investigated, which can be considered as elementary step in long range charge transport. In solution, electron uptake is accompanied by protonation of the resulting hypothetical anion **3·**, which leads to leucoverdazyl **3H** and a marked structural change, thereby excluding such reductions in densely packed solid state structures. Electron donation on the other hand proceeds with only small structural changes, which leads to low contributions of *λ_i_* to the activation barrier for ET reactions according to Marcus’ theory, which was also confirmed by DFT calculations. However, EPR line broadening experiments suggest that the favorably low value of *λ_i_* is partially counterbalanced by other contributions to the activation energy for ET. One possible source for this counterbalancing might be *λ_o_*, which is suspected to be larger for **3·**/**3^+^** as compared to the TEMPO/TEMPO^+^ redox couple. In the solid state, **3^+^**[BF_4_]^−^ showed semiconductor behavior, albeit only with low conductivities. The in general lower oxidation potential of Kuhn verdazyls might render them superior electron donors as compared to oxoverdazyls [34].

Finally, it is remarked that achieving strong enough magnetic interactions for real-life applications in MMMs remains challenging without implementing transition metal ions (TMI), using purely organic compounds like verdazyls, nitroxides, trityls, or other organic radicals instead. However, nitroxides and trityls have also found applications in numerous other fields [1,10], for example as spin label/spin probes in EPR spectroscopy and imaging [59,90,91,92,93,94,95,96], as polarizing agents in dynamic nuclear polarization enhanced NMR spectroscopy [97,98,99,100,101,102], in chemical synthesis [103,104] or as dopants in nanoparticles [105,106]. Owed to the in general lower toxicity of TMI free compounds, many of these applications are related to the increasingly important field of biochemistry. Similar developments with respect to their application of verdazyl radicals are conceivable, and first results have been obtained in some of the aforementioned fields [38,107,108,109,110,111,112].

## Figures and Tables

**Table 1:** Isotropic *g*-values *g_iso_*, hyperfine coupling constants *A_iso_* and peak-to-peak linewidth *LWPP* for verdazyl radicals **3·**, **5·** and **6·** as obtained from EasySpin [60] simulations of the EPR spectra.

Cmpd	*g_iso_*	*A_iso_*(N1/N2) ^a^ [MHz]	*A_iso_*(H*_N_*_-Phenyl_) ^b^ [MHz]	*LWPP*^c^ [MHz]
**3**	2.00365	16.81/16.62	3.05, 1.30, 3.30, 0.76	0.22, 0.60
**5**	2.00366	16.81/16.62	3.05, 1.25, 3.30, 0.72	0.22, 0.60
**6**	2.00366	16.81/16.62	3.05, 1.25, 3.30, 0.72	0.22, 0.60

^a^ Nitrogen atoms N4 and N3 are symmetry equivalent to N1 and N2, respectively. ^b^ The given numbers refer to the symmetry equivalent *o*-, *m*-, and *p*-protons on the *N*-phenyl rings as well as to the *o*-protons of the C3-phenyl ring, respectively. Introduction of the *m*-protons of the C3-phenyl ring did not improve the simulations. ^c^ LWPP = linewidth peak-to-peak. The two numbers refer to the Gaussian linewidth (inhomogeneous broadening) and Lorentzian linewidth (homogeneous or life-time broadening).

**Table 2:** Geometrical parameters from the crystal and DFT structures of **3·**, **3^+^**, **5·** and **6·**.

Cmpd	C3N_i_N_j_CH_2_^a^ [°]	C3NNC_Ph_^a^ [°]	d(C3-N) ^b^ [Å]	d(N-N) ^b^ [Å]	d(N-CH_2_) ^b^ [Å]
**3· exp.**	22.30	168.70	1.339	1.359	1.454
**3· DFT**	21.55	166.77	1.334	1.342	1.448
**3^+^ exp.**	18.38	160.50	1.351	1.305	1.461
**3^+^ DFT**	17.11	154.73	1.345	1.296	1.458
**3H exp.**	32.61/19.54	95.69/161.11	1.396/1.290	1.410/1.399	1.454/1.456
**3H DFT**	34.27/16.15	96.10/174.96	1.398/1.289	1.401/1.362	1.465/1.441
**5· exp.**	20.97	171.14	1.340	1.342	1.459
**5· DFT**	21.48	166.48	1.335	1.341	1.448
**6· exp.**	23.34	169.76	1.337	1.366	1.461
**6· DFT**	21.51	166.06	1.335	1.341	1.448

^a^ Two chemically equivalent dihedral angles with slightly different values exist in the structure. The given value is the average dihedral angle, with the exception of **3H**, where the angles are not chemically equivalent and the first value refers to the angle involving the NH-group. ^b^ Two chemically equivalent bonds with slightly different lengths exist in the structure. The given value is the average bond length, with the exception of **3H**, where the bonds are not chemically equivalent and the first value refers to the bond involving the NH-group.

## Supplementary Materials

The following are available online: IR spectra of **2**, **3·**, **3H**, **3^+^**[BF_4_]^−^, **5·** and **6·**, band gap plots for **3^+^**[BF_4_]^−^, spin density and orbital plots for **3·**, **3H**, **5·** and **6·**, S obtained by DFT.

## Abbreviations

ET	electron transfer
MMM	multifunctional molecular materials
AF	antiferromagnetic
*J*	isotropic exchange coupling constant
TTF	tetrathiafulvalene
(BS)-DFT	(broken-symmetry) density functional theory
(cw) EPR	(continuous wave) electron paramagnetic resonance
TMS	trimethylsilyl
DMF	dimethylformamide
Asc(OH)_2_	ascorbic acid
TBAF	tetrabutylammonium fluoride
DCM	dichloromethane
HOMO	highest occupied molecular orbital
LUMO	lowest unoccupied molecular orbital
*A_iso_*	isotropic hyperfine coupling constant
LWPP	peak-to-peak linewidth
$\gamma_{e}$	gyromagnetic ratio of the electron
*k_diff_*	diffusion rate constant
*k_ET_*	rate constant of ET
*α*	proportionality constant to calculate *k_ET_*
*η*	solvent viscosity
*R*	universal gas constant
*T*	temperature
TEMPO	tetramethylpiperidine-*N*-oxide
*H_exc_*	exchange spin-Hamiltonian operator
HFX	Hartree-Fock Exchange
*χ*	magnetic susceptibility constant
LCM	linear chain model
IR	infrared
TLC	thin layer chromatography
*I*	electrical current
*U*	voltage
*σ*	conductivity
*λ*	reorganization energy
TMI	transition metal ions
