# Supplementary material for: Verdazyls as Possible Building Blocks for Multifunctional Molecular Materials: A Case Study on 1,5-Diphenyl-3-(p-iodophenyl)-verdazyl Focusing on Magnetism, Electron Transfer and the Applicability of the Sonogashira-Hagihara Reaction
Source: Molecules. 2018 Jul 18;23(7):1758. doi: 10.3390/molecules23071758 (PMC6100452; doi:10.3390/molecules23071758)
Supplement: Supplementary file 1 [file molecules-23-01758-s001.pdf]

Verdazyls as possible building blocks for multifunctional materials: A case study on 1,5-diphenyl-3-(*p*-iodophenyl)-verdazyl focusing on magnetism, electron transfer and the applicability of Sonogashira-Hagihara reaction

Hannah Jobelius, Norbert Wagner, Gregor Schnakenburg and Andreas Meyer*

*Max-Planck-Institute for Biophysical Chemistry, Am Faßberg 11, 37077 Göttingen, Germany

Supporting Information

Contents

1. **Crystal structures of 2 and 4** **S1**
2. **UV/Vis and IR spectroscopy** **S3**
3. **Bandgap Plots for $3^+[BF_4]^-$** **S5**
4. **χT vs *T* plots** **S6**
5. **Spin Density and Orbital Plots** **S7**
6. **Energies and XYZ coordinates of DFT geometry optimizations** **S10**
7. **References** **S18**

1. Crystal structures of 2 and 4

The crystal structure of compound **2** is presented in Figure S1. The structure is isomorphous to the structure of its bromo analogue **2Br**, which has been described earlier [1]. Formazan **2** is a mostly planar molecule with very small deviations from coplanarity of the phenyl ring planes from the central NNCNN structure (dihedral angles of 9.87°, 3.94°, and 13.45° between mean planes AB, AC, and AD, respectively, Figure S1a). The H atom attached to the N atom forms an intramolecular hydrogen bond to N4, which also explains the large chemical shift of this H atom in NMR spectroscopy of 15 ppm. An interesting feature of **2** are the Ni-Nj (i = 2 or 3, j = 1 or 4, the numbering is also used for verdazyls, see Figure 2 in the main text) and the Ni-C3 bond lengths of the NNCNN unit of the formazan, which are pairwisely nearly identical within the error of the measurement. This contrasts with the localized structure of the double bonds and the N-H bond in **2** shown in scheme 1 in the main text, which would suggest pronounced bond length alternation along the NNCNN moiety. The lack of bond length alternation is indicative of rapid H atom exchange between N1 and N4 [2]. The high degree of planarity of **2** allows the formation of head-to-tail stacked dimers in the crystal structure along the *a*-axis (Figure S1b).

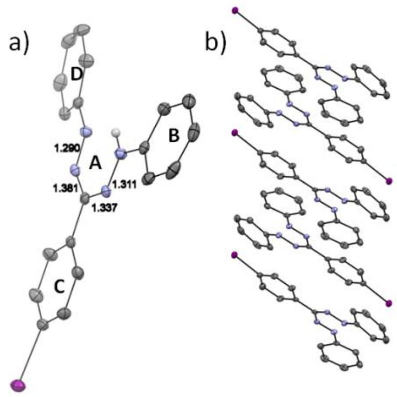

Figure S1. Molecular structures and stacking of **2** by X-ray crystallography. Ellipsoids at 50% probability level. H atoms are omitted for clarity. Color code: grey = carbon, blue = nitrogen, purple = iodine. **a)** Molecular structure of **2**, including the formazan H in white. **b)** Stacking of **2** along the *a*-axis.

The structure of by-product **4** is depicted in Figure S2a. The bond lengths within the aromatic triazole moiety of **4** lie between values expected for single and double bonds and alternate slightly, similar to observations made on a triazole side product isolated during the synthesis of **3Br**$^{\cdot}$ [1]. The molecules in the crystal structure of **4** are arranged in a fishbone pattern along the *a*-axis (Figure S2b), with a second fishbone pattern formed perpendicular to the first one (Figure S2c). No face-to-face stacking is observed in **4**, even though the molecules are almost planar (dihedral angles 15.23° and 10.99° of between the pair of rings AB and AC, respectively). One short I-C contact is observed within the first fishbone pattern (Figure S2b), which might be interpreted as weak halogen bond [3-5].

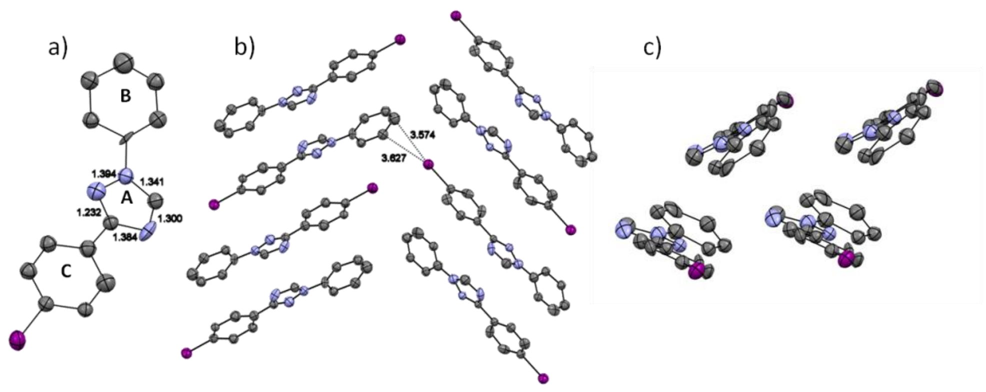

Figure S2. Molecular structures and stacking of **4** as obtained by X-ray crystallography. Ellipsoids at 50% probability level. H atoms are omitted for clarity. Color code: grey = carbon, blue = nitrogen, purple = iodine. **a)** Molecular structure of **4**. **b)** Fishbone pattern of **4** along the *a*-axis. Short I-C contacts are indicated. **c)** Second fishbone pattern formed along the *b*-axis.

2. UV/Vis and IR spectroscopy

The UV/Vis spectra of **2**, **3**$^{\cdot}$, **3**$^{+}$$(BF_4)^-$, **5**$^{\cdot}$, and **6**$^{\cdot}$ are shown in Figure S3. All compounds have intense, broad maxima in the visible region which are responsible for the typical, intense colors of the compound classes (~485 nm for formazan **2**, ~550 nm for the verdazylium salt **3**$^{+}$$(BF_4)^-$, and two maxima at ~450 and ~720 nm for verdazyls **3**$^{\cdot}$, **5**$^{\cdot}$, and **6**$^{\cdot}$). Owed to their intense color, it is usually possible to spot contaminations with any of the intensely colored compounds by visual inspection alone. The reflection IR spectra of **2**, **3**$^{\cdot}$, **3**$^{+}$$[BF_4]^-$, **3H**, **5**$^{\cdot}$ and **6**$^{\cdot}$ are shown in Figure S4. For the colorless leucoverdazyl **3H**, IR spectroscopy is a useful tool, owed to its sharp and characteristic absorption at 3310 cm^{-1}. Interestingly, **3H** is oxidized slowly enough to obtain ESI-MS signals which are clearly different from those of **3**$^{\cdot}$. Nevertheless, the spectra reveal that large fractions of **3H** are reoxidized during ionization, therefore MS might be unsuited to detect small amounts of **3H** as contaminant of the parent verdazyl **3**$^{\cdot}$.

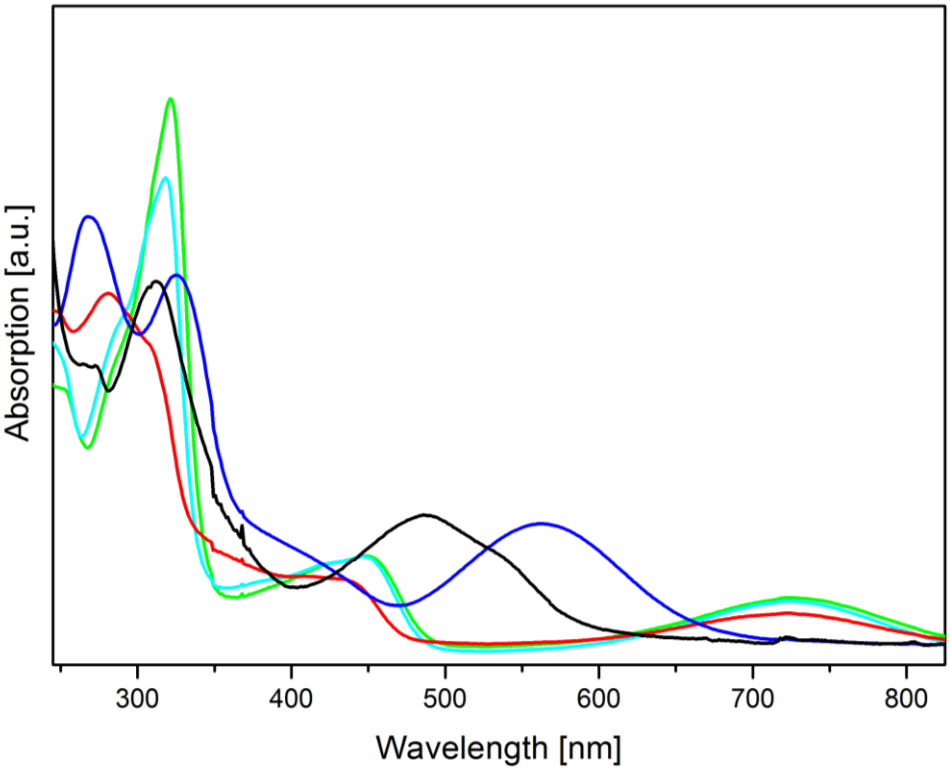

Figure S3. UV/Vis spectra of 2.3 µM DCM solutions of **2** (black), **3**$^{\cdot}$ (red), **3**$^{+}$$(BF_4)^-$ (blue), **5**$^{\cdot}$ (green), and **6**$^{\cdot}$ (cyan).

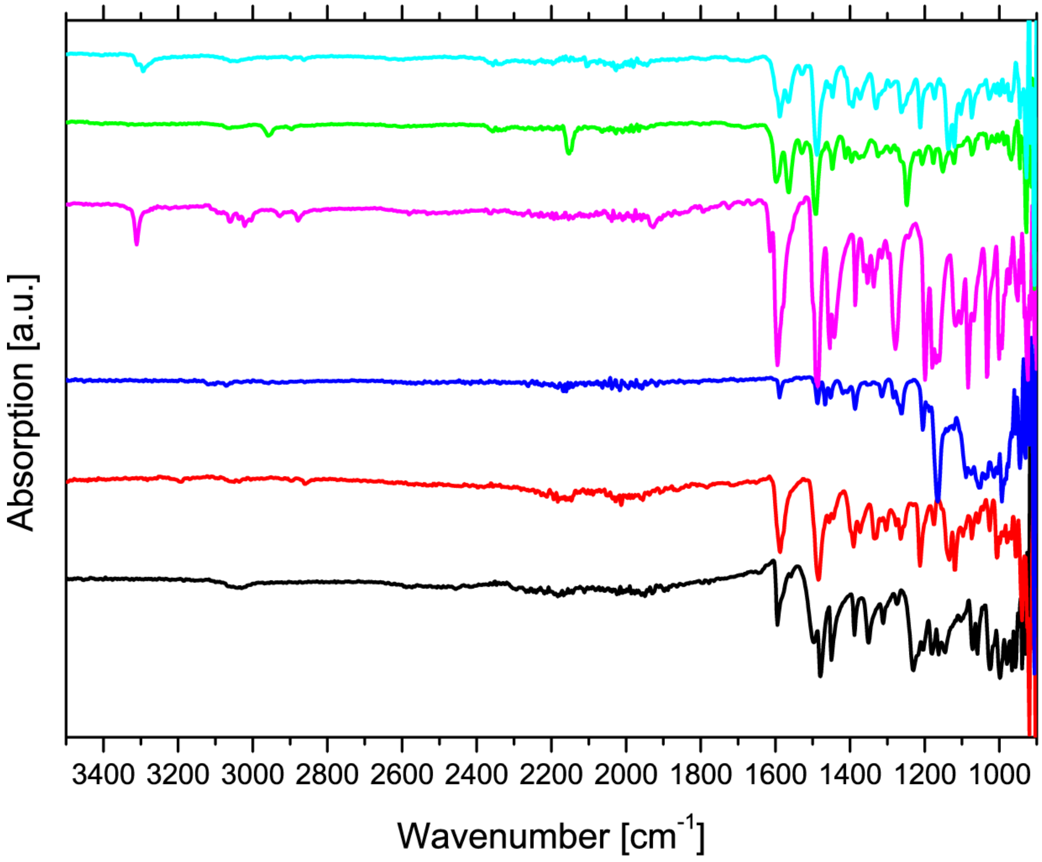

Figure S4. Reflection IR spectra **2** (black), **3**$^{\cdot}$ (red), **3**$^+$[BF$_4$]$^-$ (blue), **3H** (purple), **5**$^{\cdot}$ (green) and **6**$^{\cdot}$ (cyan).

3. Bandgap Plots for $3^{+}[BF_4]^{-}$

Figure S5 shows the plots of –ln(1/R) vs 1/T according to eq. (6) from the main text to obtain the band gap ΔE of $\mathbf{3}^{+}[BF_4]^{-}$.

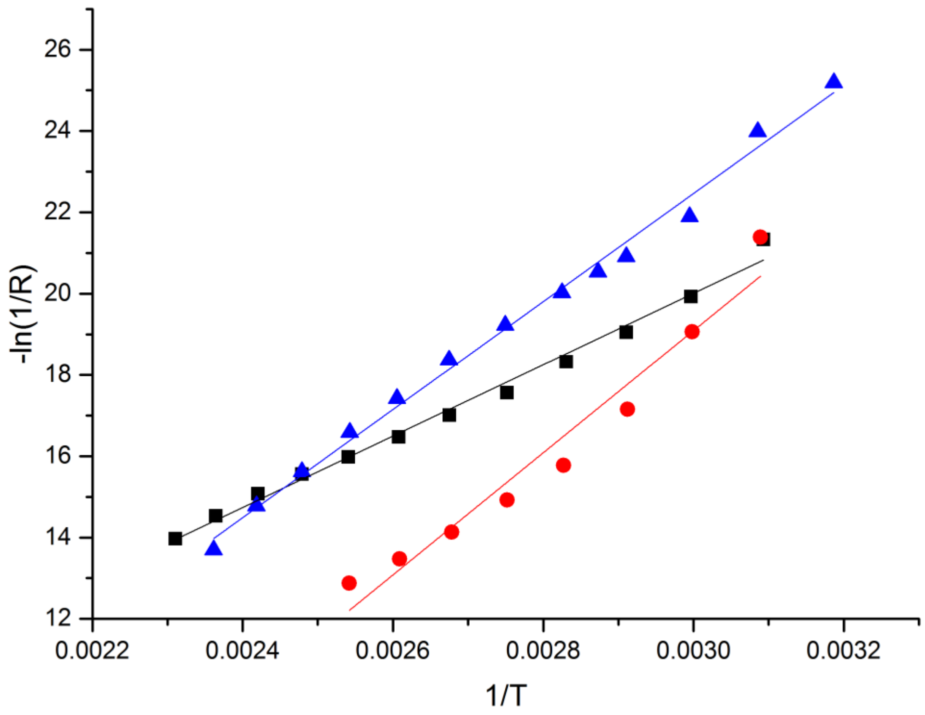

Figure S5. Plots of –ln(1/R) vs 1/T to calculate the band gap obtained on three different samples of $\mathbf{3}^{+}[BF_4]^{-}$.

4. χT vs T plots

Figure S6 shows plots of χT vs T for all verdazyls compounds presented in this work.

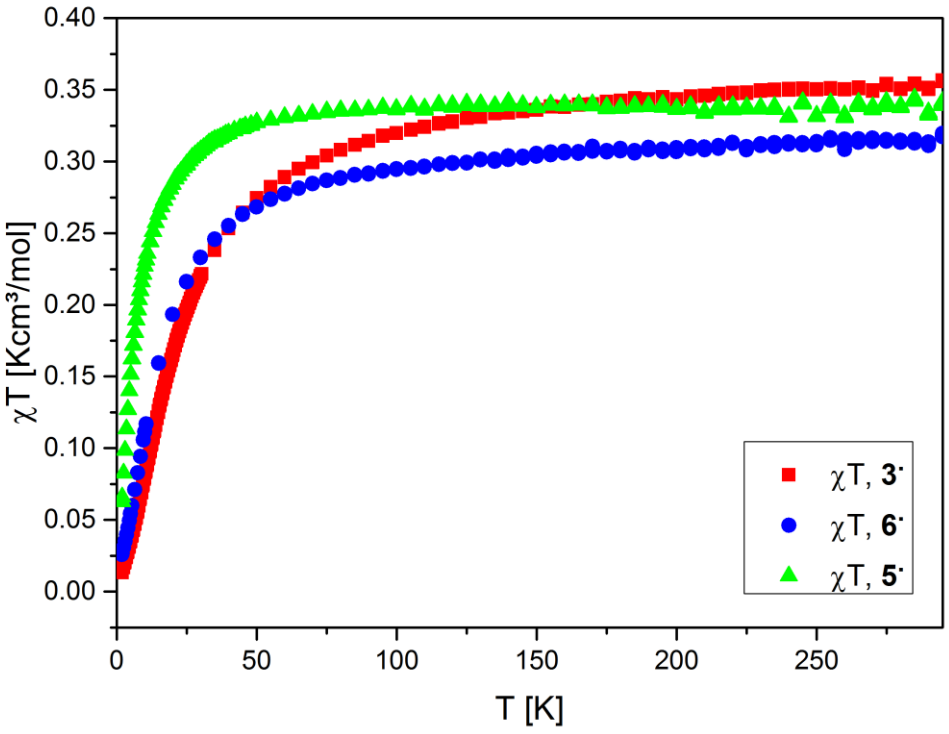

Figure S6. Plots of χT vs T for verdazyls **3**˙, **5**˙ and **6**˙.

5. Spin Density and Orbital Plots

The SOMOs and spin density plots of all verdazyls presented in the main text are practically not affected by the different substitution patterns on the 3-phenyl ring and are given in Figure S7 – S10. For the cation **3**$^{+}$, the HOMO and the LUMO have been plotted (Figures S11 and S12, note that the LUMO **3**$^{+}$ is essentially identical to the SOMO of **3**$^{\cdot}$).

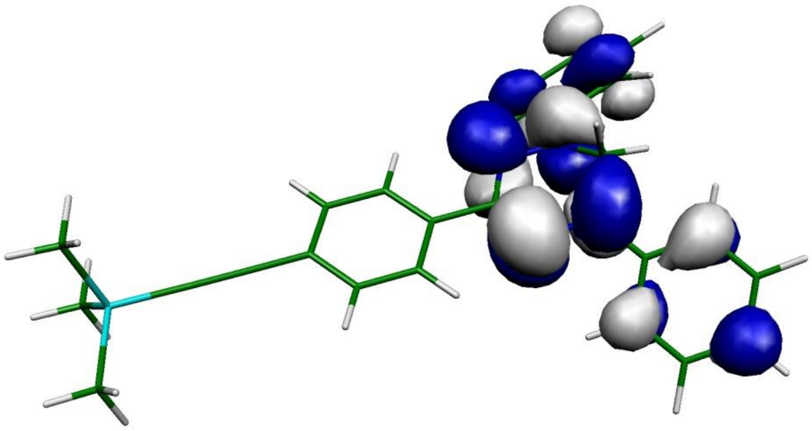

Figure S7. SOMO of **5**$^{\cdot}$.

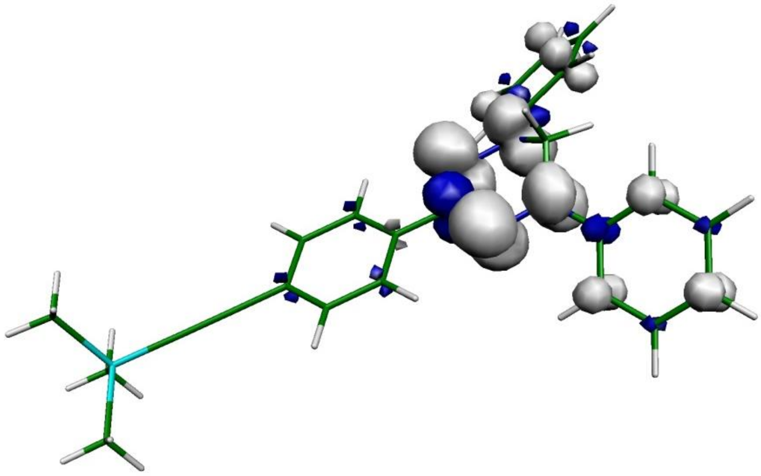

Figure S8. Spin density of **5**$^{\cdot}$.

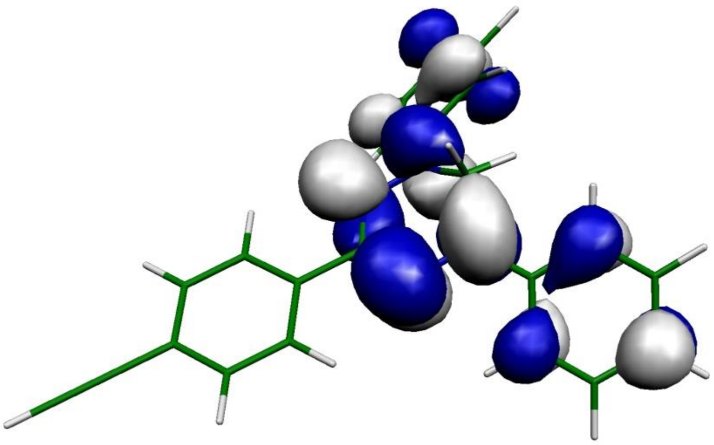

Figure S9. SOMO of $\mathbf{6}^{\cdot}$.

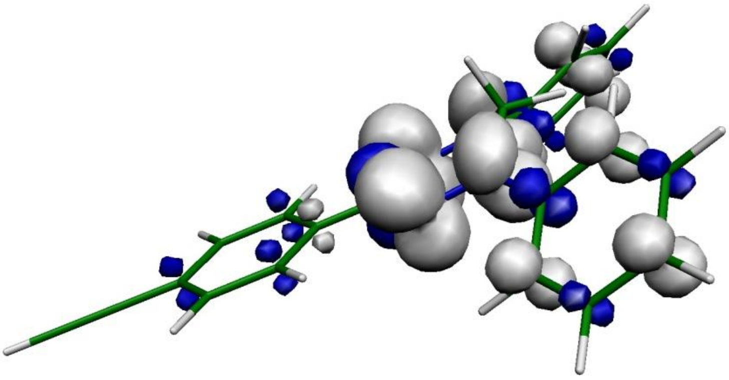

Figure S10. Spin density of $\mathbf{6}^{\cdot}$.

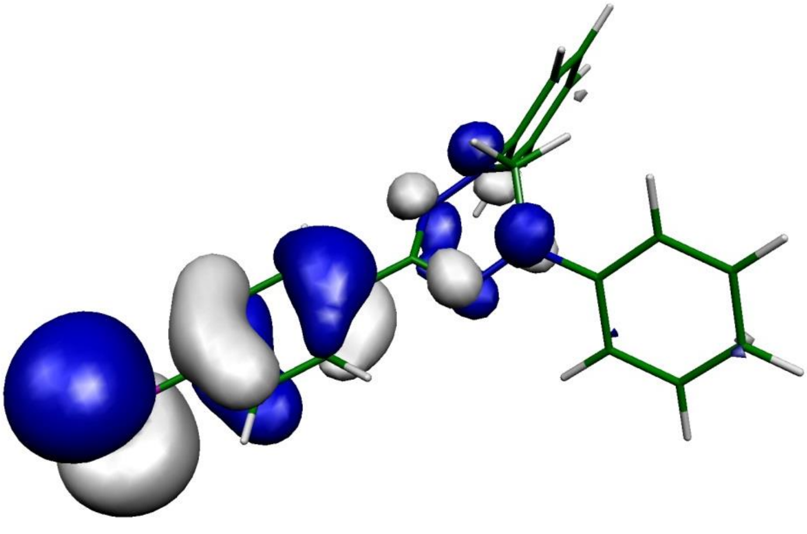

Figure S11. HOMO of $\mathbf{3}^{+}$.

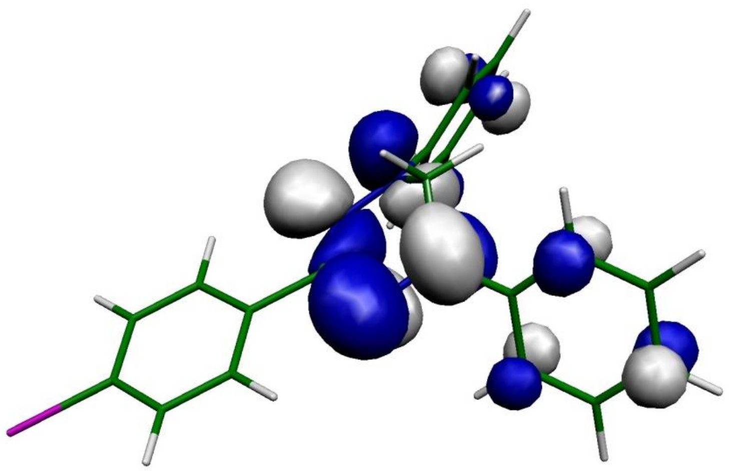

Figure S12. LUMO of **3**$^{+}$.

6. Energies and XYZ coordinates of DFT geometry optimizations

DFT geometry optimization of 3$^{\cdot}$

-7910.915611618950

XYZ:

41

C	-3.280315	0.100513	1.082169
C	-1.895265	0.149145	1.146404
C	-1.126845	0.267734	-0.013774
C	-1.780897	0.328787	-1.246175
C	-3.165382	0.280715	-1.320868
C	-3.912305	0.167392	-0.154018
H	-3.859337	0.008621	1.990453
H	-1.398379	0.092654	2.104580
H	-1.195972	0.413121	-2.151117
H	-3.655620	0.329441	-2.283271
C	0.348612	0.321422	0.060361
N	0.898378	0.288412	1.275738
N	1.008434	0.503558	-1.085147
N	2.345846	0.434477	-1.003868
N	2.238068	0.225487	1.307692
C	2.910968	-0.273203	0.125954
H	2.747429	-1.355512	0.021587
H	3.969850	-0.059906	0.193648
C	3.111695	0.886112	-2.083100
C	4.426342	0.446059	-2.276237
C	2.551792	1.792383	-2.992368
C	5.167750	0.922405	-3.349109
H	4.870949	-0.286335	-1.617025
C	3.302058	2.249663	-4.062108
H	1.537419	2.126713	-2.838296

C	4.615152	1.825191	-4.247990
H	6.181878	0.569931	-3.486536
H	2.858340	2.954012	-4.754685
H	5.196301	2.191382	-5.083802
C	2.896488	0.467447	2.517432
C	4.201251	0.012055	2.739396
C	2.236806	1.175501	3.530270
C	4.835663	0.282006	3.944555
H	4.721591	-0.573234	1.994555
C	2.880186	1.425830	4.730071
H	1.230151	1.521634	3.354024
C	4.184177	0.988608	4.946818
H	5.844796	-0.076855	4.100546
H	2.359588	1.977887	5.502541
H	4.682444	1.193679	5.884944
I	-6.023606	0.085735	-0.261922

DFT geometry optimization of 3H

-7911.528555850694

XYZ:

42

C	-3.10701066872706	0.15922786104872	1.28074750609514
C	-1.71879778660164	0.21583910416144	1.27100235205241
C	-1.00771500994233	0.19287545220355	0.06788470853316
C	-1.72379335793503	0.11234604893059	-1.13142232000178
C	-3.10735028320179	0.06653233153728	-1.13113799626677
C	-3.79777704010678	0.08572919197832	0.07870776372906
H	-3.64069014724727	0.18357687975458	2.22057179217412
H	-1.18949831375062	0.30796096352672	2.21012629405522
H	-1.17742142299138	0.08591574909574	-2.06397748587979
H	-3.64521793326545	0.00657910849936	-2.06703566744069

C	0.46061201610455	0.24669745848873	0.05068539411669
N	1.11350586348873	-0.05539930701020	1.24939816844796
N	1.06532158761483	0.62023993203689	-1.02504522809675
N	2.42538536862461	0.54979770578396	-1.05526253289082
N	2.50633582630817	0.09022725687134	1.26951246951183
C	3.05933709833753	-0.25872602369172	-0.04130426148525
H	2.86479365348748	-1.32417628670345	-0.20426859355407
H	4.12768891982359	-0.08260146052600	-0.04750589992183
C	3.07882793747528	1.05526218524898	-2.17984690000902
C	4.37963673173372	0.65305718686563	-2.50801799257752
C	2.43428261737874	1.99165168107533	-2.99923243171997
C	5.02148332342246	1.19502065472429	-3.61442072590771
H	4.89203050417497	-0.09837115683958	-1.92424507149981
C	3.08419921780339	2.51156270565362	-4.10621270091233
H	1.43229120106098	2.30243059450621	-2.74766686828491
C	4.38367764990130	2.12564025516202	-4.42308614027961
H	6.02686543934583	0.86924410693984	-3.84949439776788
H	2.56970023155640	3.23650302741573	-4.72509497678126
H	4.88575932456579	2.53991530873157	-5.28702623198918
C	2.94992510692839	1.34398262583608	1.79497943941189
C	4.31790281559737	1.52395441011321	2.01142399490891
C	2.06966565671179	2.37334152429170	2.11896103163031
C	4.79495252764541	2.72082477570993	2.52360856943066
H	5.00746465106058	0.71775992118260	1.79750506637540
C	2.55602367196684	3.56631512578677	2.64258271481843
H	1.00987826635626	2.24171309766766	1.96214561450055
C	3.91695261886479	3.75173544985201	2.84233747670969
H	5.85854462498243	2.84291956428584	2.68580942236621
H	1.85913628216196	4.35838045665613	2.88632872936370
H	4.29066529601961	4.68299382683036	3.24703638324970

I	-5.90977525428266	-0.00310404834681	0.08340727862557
H	0.80061977754822	-0.88724823533500	1.73160105316034

DFT geometry optimization of 3^+

-7910.695670488664

XYZ:

41

C	-3.39337149385003	-0.05691968258562	1.06628053724173
C	-2.01049016029022	-0.02912022114165	1.13170341073759
C	-1.24894087267293	0.08714437807037	-0.03468486655668
C	-1.89681500406247	0.15846880095015	-1.27116453991450
C	-3.27947500737696	0.13034843910041	-1.34050507997797
C	-4.02984321852920	0.02495937063423	-0.17081642607249
H	-3.97289609151000	-0.14583814236297	1.97407154757848
H	-1.51959246254407	-0.09571443789649	2.09307938657236
H	-1.31817662422321	0.23830329363544	-2.18144092865791
H	-3.77112154625993	0.18697940583906	-2.30129458802475
C	0.20984116509271	0.11381279468131	0.03550498175730
N	0.82687228317846	0.15545712134338	1.22996220394822
N	0.93267618605441	0.36592294136377	-1.07045894599734
N	2.20413284912670	0.12249273224408	-1.01778790704464
N	2.09962024556488	-0.08606354890721	1.24986657772752
C	2.64578211137781	-0.74350195183734	0.06863496509232
H	2.18820344567269	-1.72995433338104	-0.04342768046569
H	3.72362037408840	-0.79121742265271	0.11406876067370
C	3.09820990032833	0.82117788884466	-1.86841072302707
C	4.32467972304364	0.24367733671640	-2.20171209376052
C	2.71992397552682	2.06072588949339	-2.38471643799682
C	5.17871651329498	0.92245042905060	-3.05625442500141
H	4.60045905911877	-0.73592454776719	-1.83642870182881
C	3.58159676395293	2.72224997445785	-3.24316178716309

H	1.76789314941466	2.48885861646715	-2.10715849774343
C	4.81079601465476	2.15907407390275	-3.57712008577100
H	6.12712044826538	0.47900849625077	-3.32630101199018
H	3.29894594106799	3.68561256946368	-3.64458283758548
H	5.48189106923234	2.68398916724595	-4.24376499847064
C	2.90763020072162	0.43369647183059	2.29270449675846
C	4.09888036707719	-0.21277391245499	2.62600877971627
C	2.48063797789009	1.56393492668126	2.98964727090156
C	4.86876467532390	0.28543066337379	3.66471330314398
H	4.40935432694991	-1.11357928894864	2.11394647427451
C	3.25808474597839	2.04522872846070	4.02922087044729
H	1.55792361806954	2.05040346037058	2.70902896621945
C	4.45186243144256	1.41220479209011	4.36629276636927
H	5.78856797416166	-0.21442793386266	3.93518742536491
H	2.93762272467780	2.92396375305771	4.57202366557952
H	5.05655435398729	1.79709876837308	5.17641593437329
I	-6.12966336301757	-0.02697371019470	-0.27484957142731

DFT geometry optimization of 5·

-1475.928800019919

XYZ:

55

Si	11.82775327656391	-2.06923740693652	13.98956886297033
N	13.01275398082916	1.89918371629381	5.50012182937848
N	13.01058523457807	2.61431820150308	4.36534601133774
C	11.85235372483947	-0.08255997989128	9.98969311038051
C	13.04227499242943	0.06949740427199	9.26316643057307
H	13.96559651981447	-0.30973338616351	9.67961202158605
C	13.04176592518720	0.69942238715118	8.03177653069143
H	13.96449023177350	0.81639404443528	7.48170905637588
C	11.85515191565112	1.19518843569706	7.48295971098982

C	11.85080142163158	-0.72186527520771	11.25867302695591
C	11.84893776291401	-1.26128895815196	12.34532538938021
C	13.45295084337287	-2.97233365177689	14.23397777608918
H	14.29622221479704	-2.28041131479314	14.18222892592011
H	13.47847368661028	-3.46350779799984	15.21024103256094
H	13.59716861005535	-3.73627823176872	13.46722604213191
C	10.39287894767283	-3.27557270525914	14.04489572903844
H	10.49277918302632	-4.04170408379868	13.27304560860720
H	10.34495114961513	-3.77694888312381	15.01526791036138
H	9.44305454295939	-2.76066809551809	13.88480180780853
C	11.85643104603933	1.86657927271759	6.16702362737201
C	11.98585429898658	3.62600846122352	4.21494489749643
H	11.95735436539649	3.96272551754826	3.18798411891920
H	12.18385605143945	4.47338712678984	4.88631361859131
C	14.07375806263948	2.46472362644105	3.46942292631315
C	14.86488888083560	1.30989880257776	3.52650667892544
H	14.63374144232099	0.55585571703109	4.26310072215090
C	15.91711327670677	1.14885374294103	2.64121521740083
H	16.51640544515803	0.24858452638036	2.69360732745742
C	16.20190239100334	2.11978634123528	1.68418341033965
H	17.02333086422837	1.98439550108809	0.99322284362216
C	15.42084853937661	3.26722226289804	1.63226040151893
H	15.63735281515323	4.03958095954131	0.90499561335402
C	14.36792575790103	3.44991736103602	2.51879871596386
H	13.80526829841811	4.37193369590891	2.47877065059539
N	10.68121107973753	2.29453234013911	5.70037344368528
N	10.72699669613483	2.99838022268071	4.55969680777921
C	10.66411665918757	0.41832505977624	9.43817378255369
H	9.74031593269075	0.30944679226012	9.99040477399143
C	10.66783866619505	1.04808128742889	8.20688047312868

H	9.74888393287268	1.43642812487809	7.79256127692296
C	11.61317003924484	-0.74833291132077	15.30373086056067
H	12.43252214945758	-0.02667878084022	15.27368059768693
H	11.59156567715825	-1.19426172042938	16.30175923524187
H	10.67938479898179	-0.20038387107283	15.15903359009410
C	9.53680291401524	3.23656212926659	3.86557722821689
C	8.41789028680222	2.43309282543609	4.12291675467174
H	8.50059434263540	1.64294367613059	4.85330967569490
C	7.23402984016946	2.65653892369979	3.44084068387590
H	6.37913821071644	2.02543972808752	3.64901625712873
C	7.13628070922413	3.66900517725967	2.49016053136193
H	6.20947236102835	3.83435492598872	1.95739216178881
C	8.24435513516125	4.46735790098892	2.23809812311926
H	8.18343217091843	5.26822863568933	1.51196284711587
C	9.43626613778872	4.26521914481993	2.92114959005130
H	10.26677255995521	4.92869305481181	2.72755872216797

DFT geometry optimization of 6·

-1067.278833866947

XYZ:

43

N	3.66622227219852	9.57882123909203	2.42081220370307
N	2.58971568689994	10.33483379151573	2.15922102676682
N	3.82553857608754	12.27746579563651	2.47300529844257
N	4.92669341313960	11.56317340239696	2.74739736658349
C	10.57241816228287	6.47947690808318	3.29975464938089
H	11.46082430035030	5.90670847666095	3.40347645014451
C	9.56688836195691	7.12960080621090	3.18247323024766
C	8.37920375288207	7.90283422281156	3.04920182686398
C	8.42561320331511	9.30165909628027	3.12131033073551
H	9.37637833574300	9.79007360569648	3.28716067996481

C	7.27346093910544	10.05360570215712	2.97795635742205
H	7.31915192729739	11.13224921647560	3.02845919827285
C	6.03850216587720	9.43485963367655	2.76150824656130
C	5.99034628598251	8.03932198354776	2.69027147788789
H	5.04077221014416	7.55430237088933	2.51660403383751
C	7.14035734304361	7.28397954461075	2.83356993858042
H	7.09246347478314	6.20453622065116	2.77570827996350
C	4.81131424149569	10.24138960394785	2.59963726527759
C	2.82461873922599	11.66125137651765	1.62725516108189
H	1.91106573762931	12.23850242376010	1.66576022681799
H	3.18853686830911	11.59542548947042	0.59216018704727
C	1.31941097219559	9.75731552309973	2.24997448814456
C	1.15511025933185	8.56667824739736	2.97012493352153
H	2.01626968077968	8.11980857583490	3.44183255796895
C	-0.09799223711405	7.98683600068309	3.07177208322875
H	-0.20863257248780	7.06752531955811	3.63253161084959
C	-1.20913813520403	8.57515505623846	2.47359864999679
H	-2.18618068388203	8.11946132267342	2.56269661681120
C	-1.04503632983906	9.75314414817645	1.75676158362236
H	-1.89497656900239	10.21900924971237	1.27508129931280
C	0.20660121291857	10.34135796115513	1.63288832148031
H	0.30367956855658	11.23766961037006	1.03813297875793
C	3.76608225504233	13.61375567812973	2.87974540890214
C	2.89221523916960	14.52146760826425	2.26968521206157
H	2.26909682877057	14.22247383465732	1.43853145168861
C	2.84191389029662	15.83851745490665	2.70620209042902
H	2.16189335512952	16.52793531115084	2.22217433489654
C	3.66271610421646	16.27627657745552	3.73743766572824
H	3.61923694828898	17.30364680963922	4.07365311432923
C	4.54390889329362	15.37591653307159	4.33026687803924

H	5.18997655999190	15.70238557643957	5.13556819785205
C	4.60033419272995	14.05660090109656	3.91411570711205
H	5.27432056906818	13.35312279020090	4.37881637968300

7. References

1. Schnakenburg, G.; Meyer, A. Syntheses, Spectroscopy, and Crystal Structures of 3-(4-Bromophenyl)-1, 5-Diphenylformazan and the 3-(4-Bromophenyl)-1, 5-Diphenylverdazyl Radical and the Crystal Structure of the by-Product 5-Anilino-3-(4-Bromophenyl)-1-Phenyl-1H-1, 2, 4-Triazole. *Acta Cryst. Sect. E* **2018**, *74*, 292–297.

2. Buemi, G.; Zuccarello, F.; Venuvanalingam, P.; Ramalingam, M.; Ammal, S. S. C. Ab Initio Study of Formazan and 3-Nitroformazan. *J. Chem. Soc. Faraday Trans.* **1998**, *94*, 3313–3319.

3. Schollmeyer, D.; Shishkin, O. V.; Rühl, T.; Vysotsky, M. O. OH–π and Halogen–π Interactions as Driving Forces in the Crystal Organisations of Tri-Bromo and Tri-Iodo Trityl Alcohols. *CrystEngComm* **2008**, *10*, 715–723.

4. Gilday, L. C.; Robinson, S. W.; Barendt, T. A.; Langton, M. J.; Mullaney, B. R.; Beer, P. D. Halogen Bonding in Supramolecular Chemistry. *Chem. Rev.* **2015**, *115*, 7118–7195.

5. Metrangolo, P.; Meyer, F.; Pilati, T.; Resnati, G.; Terraneo, G. Halogen Bonding in Supramolecular Chemistry. *Angew. Chem. Int. Ed.* **2008**, *47*, 6114–6127.
